# Procognitive Potential of Neuroprotective Triazine 5-HT_6_ Receptor Antagonists Tested on Chronic Activity In Vivo in Rats: Computer-Aided Insight into the Role of Chalcogen-Differences on the Pharmacological Profile

**DOI:** 10.1021/acschemneuro.4c00873

**Published:** 2025-02-28

**Authors:** Magdalena Jastrzębska-Więsek, Sabrina Garbo, Agnieszka Cios, Natalia Wilczyńska-Zawal, Anna Partyka, Ewelina Honkisz-Orzechowska, Ewa Żesławska, Jarosław Handzlik, Barbara Mordyl, Monika Głuch-Lutwin, Alessia Raucci, Marius Hittinger, Małgorzata Starek, Monika Dąbrowska, Wojciech Nitek, Tadeusz Karcz, Alicja Skórkowska, Joanna Gdula-Argasińska, Kinga Czarnota-Łydka, Patryk Pyka, Ewa Szymańska, Katarzyna Kucwaj-Brysz, Clemens Zwergel, Anna Wesołowska, Cecilia Battistelli, Jadwiga Handzlik

**Affiliations:** † Department of Clinical Pharmacy, Jagiellonian University, Medical College, 9 Medyczna Street, 30-688 Kraków, Poland; ‡ Department of Molecular Medicine, Sapienza University of Rome, Viale Regina Elena 324, 00161 Rome, Italy; § Department of Technology and Biotechnology of Drugs, Jagiellonian University, Medical College, 9 Medyczna Street, 30-688 Kraków, Poland; ∥ Institute of Biology and Earth Sciences, University of the National Education Commission, Krakow, Podchorążych 2, 30-084 Kraków, Poland; ⊥ Faculty of Chemical Engineering and Technology, Cracow University of Technology, ul. Warszawska 24, 31-155 Krakow, Poland; # Department of Pharmacobiology, Jagiellonian University, Medical College, 9 Medyczna Street, 30-688 Kraków, Poland; ¶ Department of Drug Chemistry and Technologies, Sapienza University of Rome, Piazzale Aldo Moro 5, 00185 Rome, Italy; ∇ Pharmbiotec gGmbH, Nußkopf 39, 66578 Schiffweiler, Germany; ○ Department of Inorganic Chemistry and Pharmaceutical Analytics, Jagiellonian University, Medical College, Medyczna 9, 30-688 Kraków, Poland; ⧫ Faculty of Chemistry, Jagiellonian University, Gronostajowa 2, 30-387 Kraków, Poland; †† Imaging Laboratory, Center for the Development of Therapies for Civilization and Age-Related Diseases, Jagiellonian University Medical College, Medyczna 9, 30-688 Krakow, Poland; ‡‡ Division of Bioorganic Chemistry, School of Pharmacy, Saarland University, Campus B 2.1, D-66123 Saarbrücken, Germany

**Keywords:** memory disturbances, 5-HT_6_R ligands, 1,3,5-triazine, behavioral tests

## Abstract

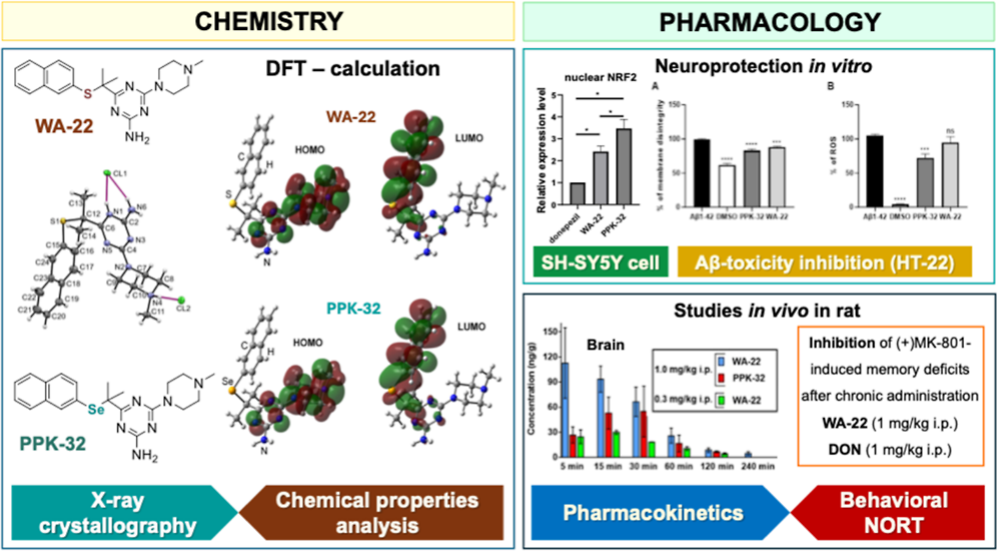

Among serotonin receptors, the 5-HT_6_ subtype is an important protein target and its ligands may play a key role in the innovative treatment of cognitive disorders. This study aimed to extend the body of preclinical research on two naphthyl-derived methylpiperazine-1,3,5-triazine analogues with thioether (**WA-22**) or Se-ether (**PPK-32**) linkers, the newly described compounds having high affinity and selectivity for 5-HT_6_ receptors and drug-like parameters in vitro. Thus, crystallography-supported deeper insight into their chemical properties, the comparison of their neuroprotective and pharmacokinetic profiles, and especially their impact on memory disturbances after chronic administration to rats were investigated. As a result, the chronic administration of **WA-22** completely reversed **(+)MK-801**-induced memory disturbances evaluated in the novel object recognition test (NORT) in rats. The pharmacokinetic and biochemical results support the notion that this 1,3,5-triazine 5-HT_6_ receptor ligand could offer a promising therapeutic tool in CNS-related disorders. The selenium compound **PPK-32**, with a similar range of activity at acute administration, has shown even broader neuroprotective profiles, especially at the genetic level. However, for therapeutic use, its weaker pharmacokinetics (stability), which is a probable limit for action upon chronic administration, would require improvement, e.g., by an appropriate formulation.

## 1 Introduction

5-Hydroxytryptamine 6 receptor (5-HT_6_R) is located almost exclusively in the central nervous system. Its localization is closely related to the brain regions responsible for learning and memory processes (i.e., dorsal hippocampus and cortex).^1,2^ It has been shown that 5-HT_6_R ligands, especially antagonists, may reverse memory impairments induced by, e.g., scopolamine or **(+)MK-801**.^3−7^

In this context, searching for potent and selective 5-HT_6_R ligands, in particular antagonists, gives a chance to develop innovative therapies for CNS diseases with memory impairment, including such important and hard-to-treat disorders as Alzheimer’s disease (AD).

Despite a lot of scientific efforts that allowed the identification of hundreds of potent 5-HT_6_R antagonists, none have reached the pharmaceutical market, and among the reasons for this lack of success is either related to poor pharmacokinetics/ADMET profile or small structural versatility as most developed compounds are limited to sulfone- and indole-containing structures.^8^

In search of structurally innovative 5-HT_6_ ligands, our research group identified and developed a series of original, nonindole, and sulfone-lacking derivatives of 1,2,3-triazine^9−13^ that displayed significant affinity and selectivity for 5-HT_6_R. They possess very promising CNS-druggability features, first identified theoretically,^13^ then confirmed in vitro^9−13^ and in pharmacokinetic rat models in vivo.^9,10^ Rational subsequent pharmacomodulation allowed the identification of several hits, which, apart from very potent antagonistic action on 5-HT_6_R, demonstrated a beneficial safety and ADMET profile as well as procognitive ability in the range of the current-approved AD-agent donepezil, when tested in behavioral studies in rats. In the preclinical studies conducted so far for the group of triazine derivatives, including acute administration in rats, two β-naphthyl derivatives with dimethyl-branched linker containing sulfur (**WA-22**) or selenium (**PPK-32**) in the form of an ether, have come to the fore (Table 1).^10,11^

**Table 1:**
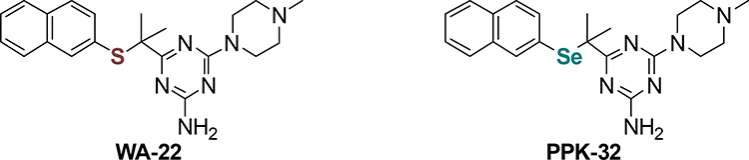
Structures and a Comparison of Pharmacological and ADMET Profiles for **WA-22** and **PPK-32** Based on the Previous Study Results^10,11^

biological activity assay		**WA-22**	**PPK-32**
aradioligand binding assay K_i_ (nM)	5-HT_6_R	11	14
	5-HT_1A_R	2291	3533
	5-HT_2A_R	39	35
	5-HT_7_R	607	1449
bfunctional assay p*K*_B_	5-HT_6_R	7.96	7.82
permeability PAMPA in vitro	*P*_e_ × 10^–6^ cm/s	13.8	6.79
	crelative *P*_eC_	0.91	2.88
dmetabolic stability in vitro	rLMs	74%	37%
safety in vitro toxic effects	ehepatotoxic	>25 μM	>100 μM
	fmutagenic	no	no
NOR test in vivo in rat	gactive dose mg/kg i.p	0.3; 1; 3	0.3; 3

a Performed in cloned receptors.

b cAMP assay in cloned receptors in vitro.

c Caffeine used as a permeable reference, relative *P*_eC_ = *P*_e_/*P*_e_ of caffeine in the assay.

d Metabolic stability estimated as % of remaining structure observed in LC/MS after biotransformation in rat liver microsomes (rLMs).

e Hepatotoxicity tested as statistically significant HepG2 viability decrease caused by the compounds at concentrations 0.1–100 μM.

f Mutagenic risk evaluated in the AMES test.

g Dose of a compound that in a statistically significant manner reversed memory disturbances caused by MK-801 in the novel object recognition (NOR) test in rats;^12,13^ active dose for donepezil was 1 mg/kg.

Both compounds showed strong affinities for 5-HT_6_R selectivity over 5-HT_1A_ and 5-HT_7_R, with intrinsic antagonistic activity, while their significant affinity also for 5-HT_2A_R (confirmed as the antagonistic mode for **WA-22**) may constitute a dual action desirable for CNS-disease therapy. Both also demonstrated beneficial membrane permeability in the range of caffeine regarding **WA-22** or approximately 3-fold higher regarding **PPK-32** in the PAMPA model, while the metabolic stability in rLMs was 2-fold higher in the case of the *S*-ether (**WA-22**). In contrast, the Se-ether (**PPK-32**) was significantly safer in the hepatotoxicity assay in a HepG2 cell line model, while both compounds did not cause any mutagenic risk in the Ames test.^10,11^ For those reasons, both **WA-22** and **PPK-32** were promoted to behavioral assays in order to evaluate their ability to reverse memory disturbances caused by the NMDA antagonist ((+)**MK-801**) in the novel recognition object test (NORT) after acute administration in male Wistar rats. The compounds turned out very active in that test, causing statistically significant memory-protective action at doses as low as 0.3 mg/kg and being more potent than donepezil.^10,11^ In particular, **WA-22** was active at all of the tested doses (Table 1).

The promising results after a single administration of 1,3,5-triazine derivatives **WA-22** and **PPK-32** and their impact on cognitive impairments in rats prompted further experiments to be conducted to gain a broader understanding of their procognitive potential. Furthermore, the results of our studies so far indicate that the minimal structural difference, which is the replacement of sulfur with selenium, significantly differentiates the pharmacodynamic and pharmacokinetic profile and the safety of these compounds. This intriguing issue seemed to require in-depth analysis at a molecular level.

Hence, the goal of the present study was to extend the body of preclinical research on **WA-22** and **PPK-32**, including crystallography-supported deeper insight into their chemical properties, the comparison of their neuroprotective and pharmacokinetic profiles, and especially their impact on memory disturbances after chronic administration in rats.

In this context, crystallographic analysis for **WA-22**, to support a comprehensive computer-aided estimation of physicochemical properties relevant to pharmacological activities of both **WA-22** and **PPK-32**, was performed in the first step of this research. Then, extended neuroprotective effects were evaluated in both neuroblastoma and HIPP cell models in vitro, and antioxidant property assays were conducted. In the next step, following intraperitoneal (i.p.) administration, the pharmacokinetic properties of **WA-22** were characterized by rapid absorption in the rat, a good distribution to the brain comparable to the results for **PPK-32** obtained previously.^10^ Thus, this study is the first report of a thorough pharmacokinetic profile and distribution characteristics of the **WA-22** compound in rats. Afterward, the ability to restore recognition memory impaired by **(+)MK-801** after chronic (21 days) i.p. administration of **WA-22** and **PPK-32** alone and **WA-22** jointly with donepezil in rats was assessed using NORT.

## 2 Results

### 2.1 Chemistry

#### 2.1.1 X-ray Crystallography Analysis for **WA-22**

In order to support the theoretical insights into the molecular properties of both triazine compounds, we managed to perform experimental crystallographic analysis for **WA-22**, while the Se compound has not given crystals suitable for this structure determination.

**Figure 1:**
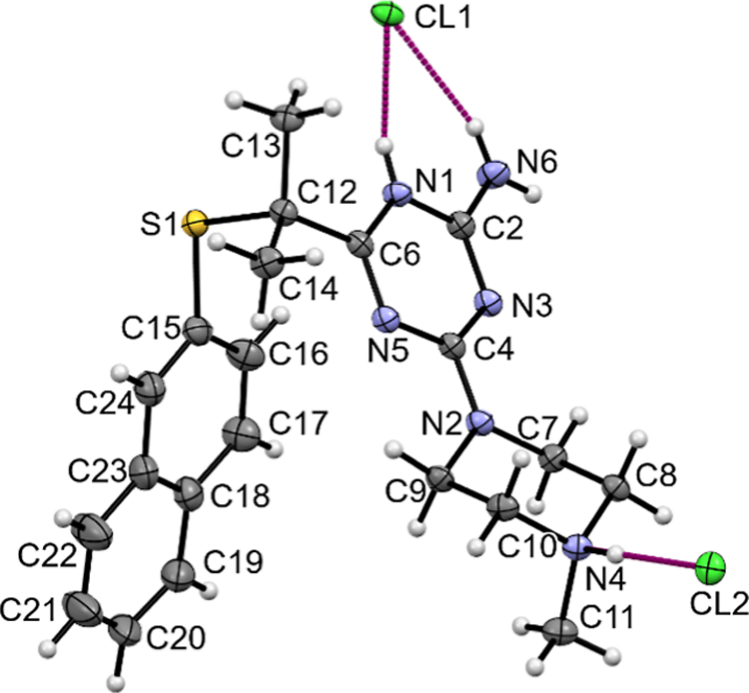
Molecular structure of **WA-22** showing the atom-numbering scheme. The purple dashed lines indicate hydrogen bonds. Displacement ellipsoids are drawn at the 50% probability level.

The projection of the molecular geometry in the crystal of **WA-22** with the atom-numbering scheme is presented in Figure 1. The molecule is double protonated, at the N1 and N4 atoms, by the proton transfer from two hydrochloride molecules. The protonated N atoms are involved in the charge-assisted N^+^-H···Cl^–^hydrogen bonds (Figure 1).

The nitrogen atoms at C2 and C4 are coplanar with a triazine ring. An analysis of the C–N bond lengths (C2–N6 and C4–N2) shows values of 1.321 and 1.331 Å, indicating a conjugation of these atoms with the triazine ring. The piperazine ring adopts chair conformation with the equatorial position of a methyl group, wherein the N2 atom shows almost the hybridization sp^2^ with the distance from the plane (C4, C7, and C9) being 0.008 Å. The flatter geometry around the N2 atom makes this part of the molecule more rigid. This phenomenon is also observed in other molecules containing a 4-(4-methylpiperazin-1-yl)-1,3,5-triazine moiety with a crystal structure available.^6,11,12,14^

**Figure 2:**
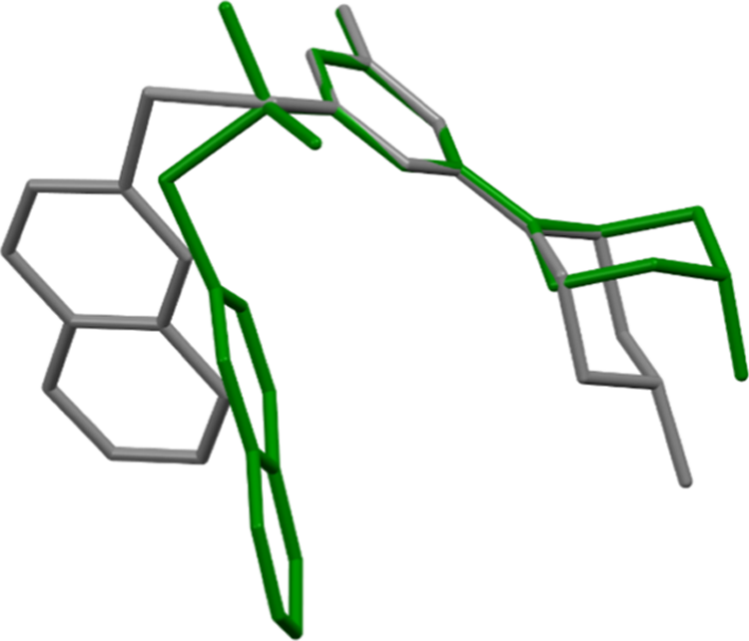
Overlap of the triazine rings of **WA-22** (green) and compound containing hydrogen atoms instead of methyl groups at the C12 atom (gray) for which the crystal structure was determined earlier.^11^ H atoms have been omitted for clarity.

We have compared the geometry of **WA-22** with a similar derivative containing hydrogen atoms instead of methyl groups at the C12 atom (Figure 2), for which we have determined the crystal structure earlier.^11^ The geometry of the linker changes as a result of the introduction of methyl groups, which causes a change in the mutual orientation of the naphthalene and triazine rings. The angles between the planes containing these rings are 38.43(6) and 64.65(3)°, for **WA-22** and compared structures, respectively. Some differences are also observed in the mutual orientation of the triazine and piperazine rings, which is best illustrated by the values of the torsion angle C4–N2–C9–C10 being −125.0(2) and 136.3(1)°, for **WA-22** and compared structure, respectively. The comparison of selected bond lengths and angles is listed in Table 2.

**Table 2:** Comparison of Selected Bond Lengths [Å], Bond Angles [°], and Torsion Angles [°] for the Presented Crystal Structure of **WA-22** and Another Derivative Published Earlier^11^

	**WA-22**	compared derivative [4]
S1–C15	1.782(2)	1.771(1)
S1–C12	1.863(2)	1.800(1)
C15–S1–C12	104.24(7)	103.68(6)
N1–C6–C12–S1	78.0(1)	38.7(2)
C6–C12–S1–C15	55.2(1)	52.4(1)
C4–N2	1.331(2)	1.336(2)
C9–N2–C4	123.0(1)	122.6(1)
C4–N2–C7	122.7(1)	122.3(1)
C7–N2–C9	114.3(1)	114.0(1)
C4–N2–C7–C8	126.6(1)	–137.2(1)
N2–C7–C8–N4	54.7(1)	56.5(1)

**Figure 3:**
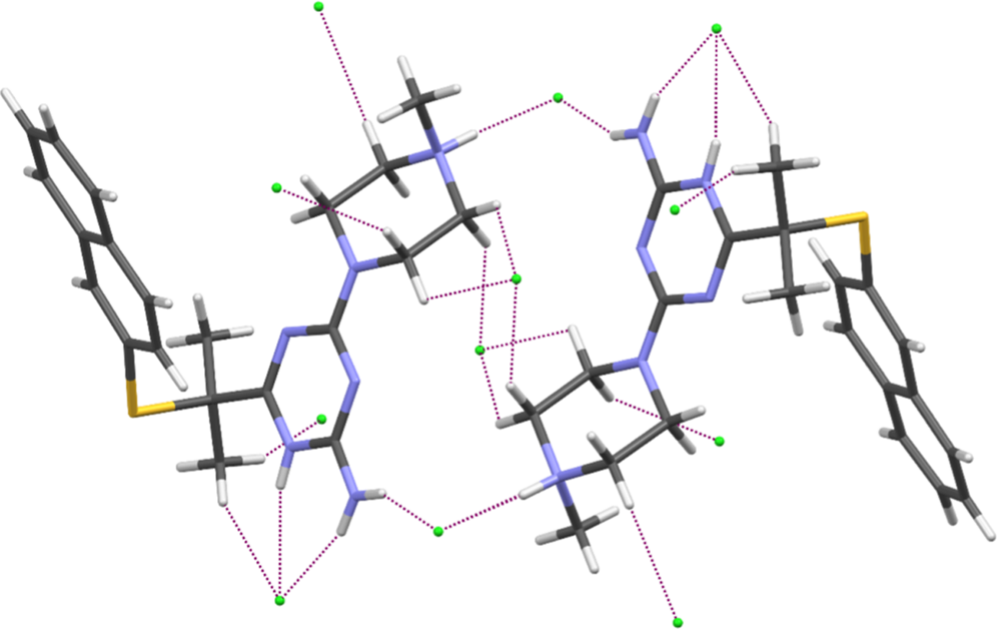
Intermolecular interactions of two molecules of **WA-22**. Dashed lines indicate the hydrogen bonds.

The main motif of intermolecular interactions is based on the ^+^N–H···Cl^–^ hydrogen bonds (Figure 3). These interactions lead to the formation of dimers. Furthermore, the crystal structure is stabilized by numerous C–H···Cl contacts.

### 2.2 Chemical Properties Analysis Based on DFT Calculation

**Figure 4:**
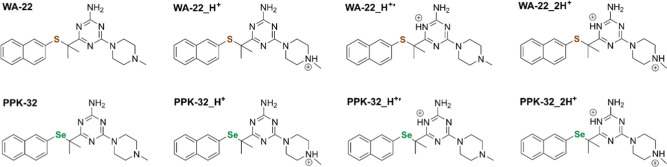
Structures of **WA-22**, **PPK-32**, and their protonated forms.

Geometries of **WA-22**, **PPK-32**, and their protonated forms (Figure 4) were optimized for the gas phase and simulated aqueous solution (Figure S1). The starting structures for geometry optimization were based on the crystallographic data for the double-protonated **WA-22_2H**^**+**^. One proton is located at the nitrogen atom of the piperazine moiety to form tertiary amine, and another one is located at the nitrogen atom of the triazine ring. We also considered the corresponding single protonated species, **WA-22_H**^**+**^ and **WA-22_H**^**+**^′, and the analogous protonated forms of **PPK-32**.

The initial conformation, where the triazine and naphthalene rings are oriented more parallel than perpendicular to each other, is maintained in most calculated geometries. The exceptions are **WA-22_H**^**+**^**′** and **PPK-32_H**^**+**^**′** in the gas phase and **WA-22_H**^**+**^**′** and **WA-22_2H**^**+**^ in water, having the triazine and naphthalene rings almost perpendicular to each other. An intermediate conformation is obtained for **PPK-32_H**^**+**^**′** in water.

Gas phase calculations suggest a higher energetic stability of **WA-22_H**^**+**^**′** and **PPK-32_H**^**+**^**′**, compared to **WA-22_H**^**+**^ and **PPK-32_H**^**+**^, respectively (Table S1). However, this trend is the opposite if the aqueous solution is simulated. Therefore, the presence of the proton at the nitrogen atom of the piperazine group is predicted to be thermodynamically more favorable than its presence at the nitrogen atom of the triazine moiety.

The calculated relative energies for the single protonated forms of **WA-22** and **PPK-32** hardly differ from each other (Table S1). It can also be noticed that the differences between the corresponding reaction energies calculated for the geometries optimized with the polarizable continuum model (PCM) model and those for the gas phase geometries are below 10 kJ mol^–1^. On the other hand, dispersion corrections (D3) affect the results obtained for the gas phase geometries more than the geometries optimized for simulated aqueous solutions. However, these differences are not very significant and can be explained by the small conformational changes mentioned above.

The relative energies of the neutral and corresponding protonated forms in aqueous solution are almost identical for **WA-22** and **PPK-32** (Table S2). An energetic preference for the protonated species in an acidic environment is predicted from the DFT calculations.

**Figure 5:**
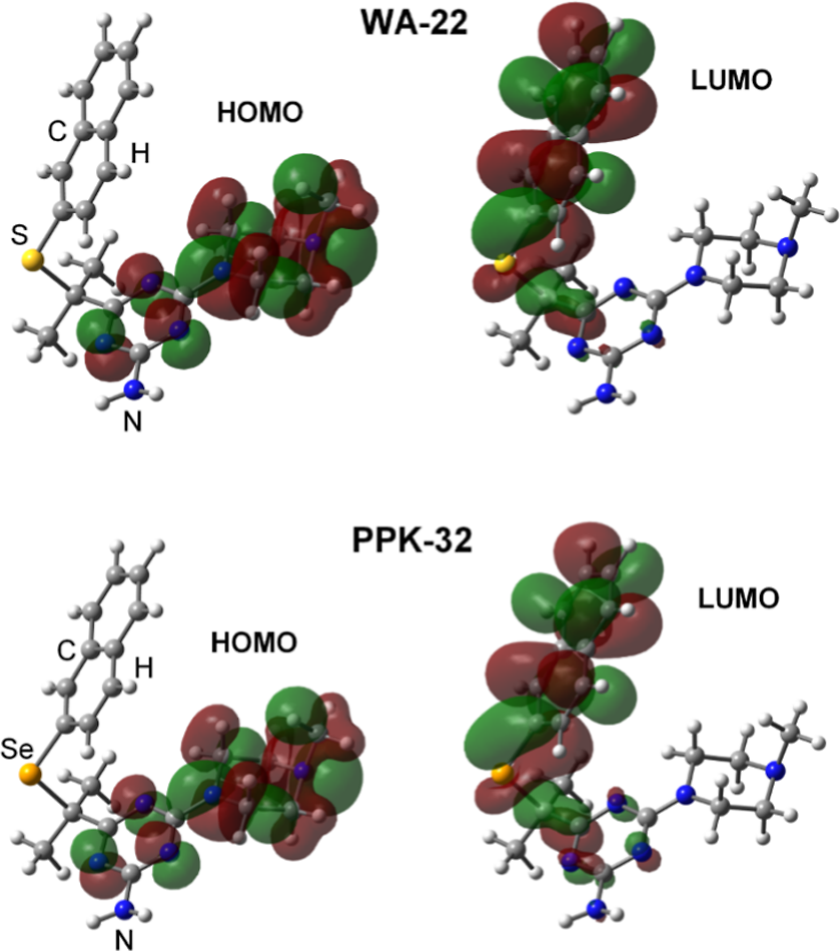
Isosurfaces of the HOMO and LUMO for **WA-22** and **PPK-32** (gas phase calculations).

For both **WA-22** and **PPK-32**, the HOMO is mainly localized on the *N*-methylpiperazine fragment, which suggests particular chemical sensitivity of this fragment in accordance with the results of previous studies with liver microsomes demonstrating the *N*-demethylated product as a main metabolite of either **WA-22** or **PPK-32** biotransformation. In contrast, the LUMO is delocalized over the naphthalene moiety and S or Se (Figure 5). These results are qualitatively the same for the gas phase and solvated molecules. The corresponding orbital energies for **WA-22** and **PPK-32** hardly differ from each other (Table 3), with the HOMO energy being slightly higher and the LUMO energy being somewhat lower in the case of **PPK-32**. The same tendency is observed for the single protonated species and for the double protonated species in an aqueous solution, but the differences in the corresponding orbital energies, although still small, are usually higher compared to the neutral molecules. In qualitative agreement with the calculated HOMO and LUMO energies, the vertical ionization potential for **PPK-32** is slightly lower than that for **WA-22** by 0.06 eV, whereas the vertical electron affinity is higher by 0.10 eV (Table 4). A similar trend is seen for the adiabatic ionization potential and adiabatic electron affinity. Consequently, **PPK-32** is characterized by a slightly more negative electron chemical potential (higher absolute electronegativity), lower hardness, and a slightly higher electrophilicity index than that of **WA-22**. Although the calculated electronic structure parameters of **WA-22** and **PPK-32** are quite similar, they may suggest a somewhat higher reactivity of the latter both as an oxidant/electrophile and, to a lesser extent, as a reductant/nucleophile.

**Table 3:** HOMO and LUMO Energy (eV) for the Neutral and Protonated forms of **WA-22** and **PPK-32**

species	gas phase		aqueous solution	
	ε_HOMO_	ε_LUMO_	ε_HOMO_	ε_LUMO_
**WA-22**	–5.15	–1.70	–5.22	–1.88
**PPK-32**	–5.13	–1.74	–5.21	–1.90
**WA-22_H^+^**	–7.88	–4.06	–5.99	–1.92
**PPK-32_H^+^**	–7.70	–4.14	–5.89	–1.96
**WA-22_2H^**+**^**	–10.27	–7.97	–6.13	–2.49
**PPK-32_2H^+^**	–10.33	–7.94	–6.11	–2.62

**Table 4:** Vertical Ionization Potential (IP_v_, eV), Adiabatic Ionization Potential (IP_ad_, eV), Vertical Electron Affinity (EA_v_, eV), Adiabatic Electron Affinity (EA_ad_, eV), Electronic Chemical Potential (μ, eV), Chemical Hardness (η, eV), and Electrophilicity Index (ω, eV) for **WA-22** and **PPK-32**

compound	IP_v_	IP_ad_	EA_v_	EA_ad_	μ	η	ω
**WA-22**	6.83	6.56	0.08	0.68	–3.45	6.76	0.88
**PPK-32**	6.77	6.53	0.18	0.84	–3.47	6.59	0.91

Geometry optimization of the radical anions of **WA-22** and **PPK-32** shows a dissociation of the S/Se–C_alkyl_ bond, in both the gas phase and aqueous solution. For the neutral compounds and their protonated forms, this bond is longer and is characterized by a lower Wiberg bond index than the S/Se–C_aromatic_ bond (Table S3). The S/Se–C_alkyl_ bond order is lower for **PPK-32** than for **WA-22**, which is also true for the corresponding protonated species. Hence, **PPK-32** may be less stable than **WA-22** and more prone to decomposition in even various reactive environments corresponding to the drug’s route from administration to reaching the therapeutic protein target in the brain. The calculated parameters of the N–CH_3_ bond, which plays a role in metabolic stability but overall forms the protonable fragment necessary for the key interaction with 5-HT_6_R, are practically identical for **WA-22** and **PPK-32** molecules in their neutral or protonated forms. This observation, in combination with the previously described extensive analysis of the receptor binding mode of both compounds in the receptor binding pocket,^10^ provides an additional explanation of the almost identical affinity of both compounds for the 5-HT_6_ receptor confirmed in vitro in RBA. Furthermore, natural population analysis (NPA) charge analysis (Table S4) confirms that replacing sulfur with selenium in the molecule does not affect the electronic structure of the *N*-methylpiperazine moiety, whereas the charges of the triazine and naphthalene fragments change little.

### 2.3 Neuroprotection In Vitro

Based on previous evidence obtained on Jurkat cells with compound **WA-22**,^11^ which significantly regulated cell cycle-regulatory genes and acted as a potent inhibitor of ABCB1, and in light of the antioxidant and neuroprotective properties of compound **PPK-32**(10) on the in vitro model of AD, the SH-SY5Y cells, a comparison analysis between the two compounds was performed. For this purpose, SH-SY5Y cells were seeded and treated with the previously used concentrations of the two compounds, 10 and 50 μM.

**Figure 6:**
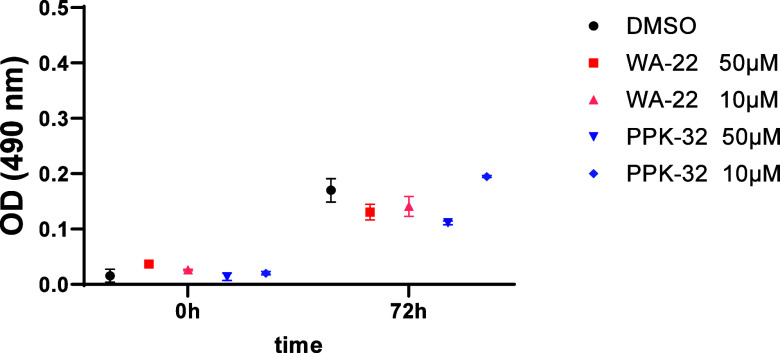
**WA-22** and **PPK-32** have no neurotoxic effect on SH-SY5Y: MTS assay on SH-SY5Y cells treated with **WA-22** and **PPK-32** at the indicated concentrations for 72 h. Data are shown as the mean ± SEM of three independent experiments.

First, the neurotoxic effect was evaluated by the MTS assay (Figure 6), and no significant differences were found at 72 h after the treatment or with the **WA-22** or the **PPK-32**.

#### 2.3.1 Neuroprotection in SH-SY5Y

##### 2.3.1.1 Cell Viability in Neuronal Cells

**Figure 7:**
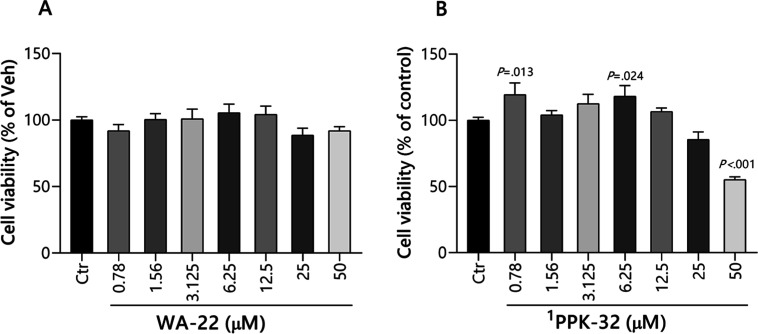
Effect of **WA-22** and **PPK-32** on human neuroblastoma cell viability. SH-SY5Y cells were incubated for 27 h with increasing compound concentrations (0–50 μM). Cell viability was measured using an MTS assay. Each point represents the mean ± SEM of three independent experiments, each consisting of three replicates per treatment group. Statistical analyses were performed using GraphPad Prism software 8.0.1. Statistical significance was evaluated by one-way analysis of variance (ANOVA) with the posthoc Dunnett test at a significance level α = 0.05. Multiplicity-adjusted *P*-values for each comparison are indicated on the graph.^1^ Data described earlier.^10^

Neurotoxicity is specific because the nervous system is crucial for transmitting and processing signals in the brain. SH-SY5Y human neuroblastoma cells present a neuroblast-like morphology and express immature neuronal markers, even though they allow the study of the mechanisms underlying the toxicity of chemical compounds on neuronal cells.^15^ In order to evaluate the potential adverse toxicity associated with innovative treatments, SH-SY5Y cells were exposed to **WA-22** in a broad range of concentrations (0–50 μM) for 27 h. The results demonstrated that the compound exhibited a safe profile toward neuronal cells, with an IC_50_ value exceeding 200 μM (Table 5). Moreover, the compound did not affect cell viability at all tested concentrations (Figure 7). **PPK-32**, despite having a lower IC_50_ value, also has a satisfactory safety profile against neuronal cells. Only at a concentration of 50 μM, **PPK-32** reduced the cell viability by 45% compared to the Ctr (*P* < 0.001).

**Table 5:** IC_50_ Values Were Determined by Fitting a Sigmoidal Dose–Response Curve to the Data Using GraphPad Prism

compound	aIC_50_ x̅ ± SD (μM)
WA-22	238.00 ± 14.98
PPK-32	b53.20 ± 4.96

a The values of IC_50_ were determined by fitting a sigmoidal dose–response curve to the data using Graph Pad Prism (equation: log(inhibitor) vs normalized response variable slope) from the MTS assay in SH-SY5Y cells at 27 h of exposure.

b (Pyka et al., 2024).

### 2.4 Neuroprotective Activity against Rotenone

**Figure 8:**
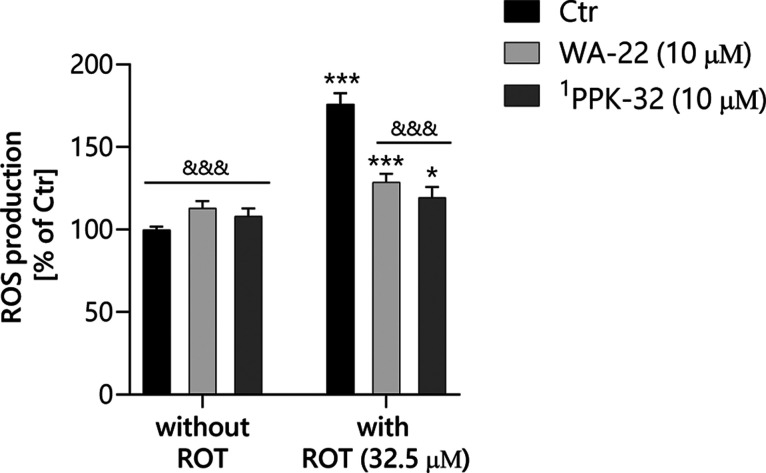
Neuroprotective effect of **WA-22** and **PPK-32** on rotenone-induced neurotoxicity evaluated through ROS production measurement. SH-SY5Y cells were pretreated with the cited compounds for 1 h, and then rotenone at a concentration of 32.5 μM was added and incubated for further 3 h. One-way ANOVA determined the significance of the difference with the posthoc Dunnett’s test (α = 0.05). *P < 0.05; ***P < 0.001 (vs control cells); &&*P* < 0.01; &&&*P* < 0.001 (vs ROT-treated cells).^1^ data described earlier.^10^

Rotenone (ROT) inhibits complex I in the mitochondrial electron transport chain.^16^ The neurotoxic effects of this toxin mimic specific characteristics of many neurodegenerative diseases, such as oxidative stress leading to the death of neurons.^17^ Because ROT impairs mitochondrial energy metabolism and increases reactive oxygen species (ROS), 2′7′DCFH_2_-DA was used to investigate the neuroprotective effect of **WA-22**. ROS levels can rapidly change and significantly impact cellular processes, so their levels were investigated shortly (3 h) after incubation with ROT. When ROT was administered alone, it led to an almost doubling of ROS generation relative to the untreated cells (Ctr) (*P* < 0.001) (Figure 8). **WA-22** alone did not exert any effect on neuronal cells, while in the presence of ROT, it protected these cells against oxidative stress (*P* < 0.001 vs ROT). Treatment of SH-SY5Y cells with **WA-22**, followed by treatment with ROT, led to a significant decline in ROS levels, reducing them from 176% (with ROT compared to Ctr set as 100%) to 128%. This effect was comparable to that of the **PPK-32** (data described earlier^10^), supporting the protective role of both compounds.

#### 2.4.1 Effects on the Expression of Neurodegeneration-Associated Genes in SH-SY5Y

Aiming at investigating the properties of **WA-22** to regulate the expression of genes involved in the antioxidant response and in the maturation of the beta-amyloid,^10,11^ a qRT-PCR on RNA from SH-SY5Y treated with both compounds at two different doses for 24 h was performed.

**Figure 9:**
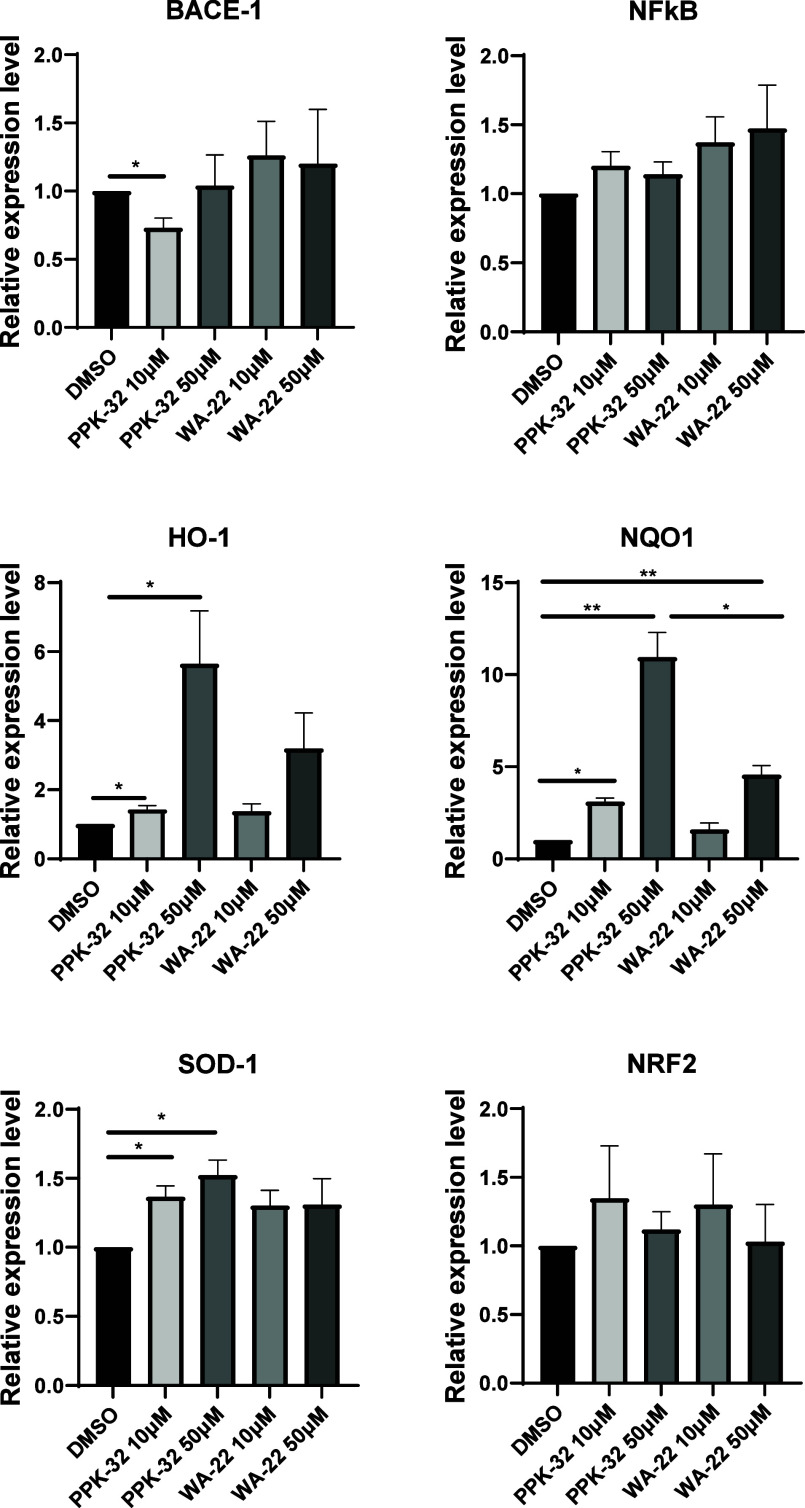
**WA-22** and **PPK-32** have an effect on the expression of antioxidant genes: expression levels of BACE-1, NFkB, HO-1, NQO1, SOD-1, and NRF2 in SHSY5Y cells treated with **WA-22** and **PPK-32** at 50 and 10 μM for 24 h. Data are shown as the mean ± SEM of four independent experiments.

As reported in Figure 9, **PPK-32** used at 10 μM was the sole compound with the ability to negatively regulate the expression of beta-secretase BACE1, responsible for the maturation of the beta-amyloid. Notably, at the tested concentrations, neither **WA-22** nor **PPK-32** were able to downregulate the expression of the pro-inflammatory gene NFkB. So, we focused on the genes involved in the antioxidant response, whose expression was previously analyzed in response to the treatment with the compound **PPK-32** at 1 and 10 μM. Data clearly indicate that **PPK-32** confirms its ability to induce the expression of NQO1, SOD1, and HO1 both at 10 μM and at the new tested dose of 50 μM. In comparison, compound **WA-22** at the same concentrations shows a certain effect. Indeed, it induces the expression of the same genes, although with a lower significance and increase, especially at a dose of 50 μM. Overall, these data confirm the effectiveness of compound **PPK-32** on SH-SY5Y cells and disclose a new activity for compound **WA-22**, whose effectiveness was previously examined in cancer cells.

**Figure 10:**
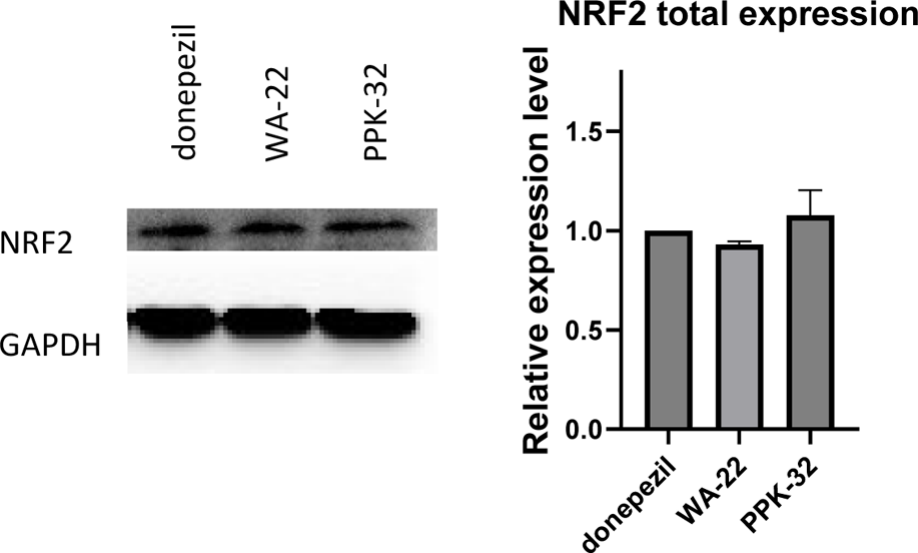
**WA-22** and **PPK-32** have no impact on the protein expression of NRF2: (Left panel) Western-blot analysis for NRF2 on protein extracts from SHSY5Y cells treated with donepezil, **WA-22**, and **PPK-32** 10 μM for 24 h. GAPDH has been used as a loading control. The figure is representative of three independent experiments. (Right panel) Densitometric analysis of Western-blot signals. Data are shown as the mean ± SEM of three independent experiments.

Because the expression of antioxidative genes can be regulated by NRF2, its expression was also assayed, but neither **WA-22** nor **PPK-32** were able to stimulate its transcription (Figure 9), and also, its protein amount was not modulated in response to the treatment with these compounds (Figure 10).

**Figure 11:**
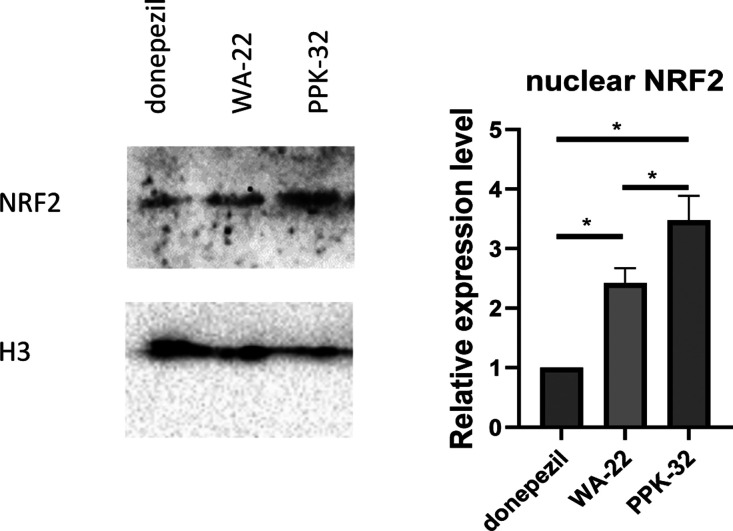
**WA-22** and **PPK-32** impact the localization of NRF2 into the nucleus: (left panel) Western-blot analysis for NRF2 on nuclear protein extracts from SH-SY5Y cells treated with donepezil, **WA-22**, and **PPK-32** 10 μM for 24 h. H3 has been used as a loading control. The figure is representative of three independent experiments. (Right panel) Densitometric analysis of Western-blot signals. Data are shown as the mean ± SEM of three independent experiments.

Thus, the effect of both inhibitors on NRF2 translocation from the cytoplasm to the nucleus was assessed. Notably, both compounds effectively induce a shuffling of this protein inside the nucleus, where it can regulate the expression of its target genes, as previously reported.^10^ Specifically, the relative expression level of NRF2 in the nuclear compartment upon **PPK-32** treatment was distinctly stronger than that upon **WA-22** treatment, and both compounds showed improved effectiveness with respect to donepezil (Figure 11).

#### 2.4.2 Neuroprotection against βA in Hippocampus Cell Line HT-22

**Figure 12:**
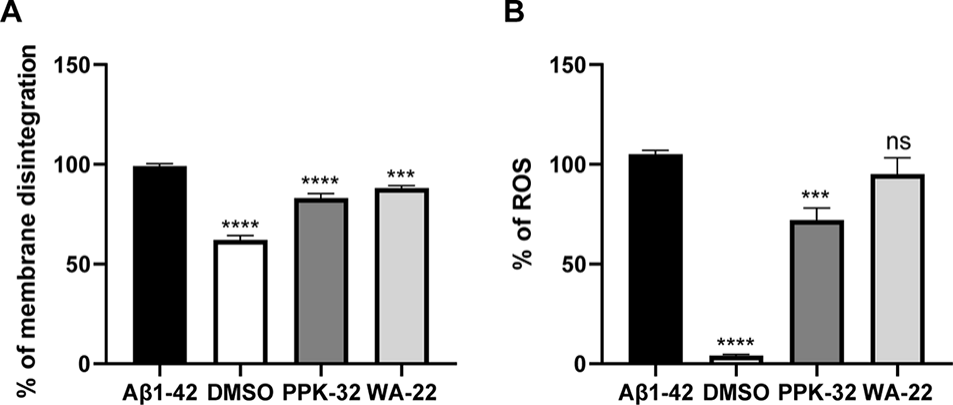
Protective effects of compounds **PPK-32** (green) and **WA-22** (purple) against Aβ-caused HT-22 cell toxicity after pretreatment with tested compounds (10 μM) or vehicle (0.1% DMSO, v/v) for 1 h; the cells were also incubated with Aβ_1–42_ in concentration 35 μM for 16 h. (A) Determination of cell membrane disintegration. (B) Determination of ROS. Data are shown as the mean ± SEM of three independent experiments. One-way ANOVA determined the significance of the difference with the ***P < 0.001; ****P < 0.0001; ns not significant.

Both **WA-22** and **PPK-32** were also investigated for their protective effects against the toxic action of Aβ on HIPP cells (HT-22 cell line). Two different assays associated with pathological processes triggered by Aβ_1–42_ in neurons were taken into account, i.e., the cell membrane disintegration and the increase of ROS. Results are shown in Figure 12.

Both compounds demonstrated neuroprotective activities in different assays. The selenoether **PPK-32** results to be slightly more potent than the thioether **WA-22** in terms of protection of cell integrity against destruction of the membrane caused by Aβ_1–42_ (Figure 12A), whereas only **PPK-32** displayed the ability to decrease ROS induced by Aβ in the HT-22 cell line (Figure 12B). The results align with those from the neuroblastoma model, where a relatively more potent protective action against ROS caused by ROT was confirmed. It is worth underlining that various neuroprotective antioxidant effects of **PPK-32** have been proven in several previous assays in vitro*.* Comparing the previous results with the present ones in hippocampal cells HT-22 and considering the impact on neurodegeneration-associated genes’ expression of both compounds, **PPK-32** seems slightly superior to **WA-22** regarding potential neuroprotective effects.

#### 2.4.3 Total Antioxidant Capacity

Considering that oxidative stress is among the primary early factors contributing to the development of neurodegenerative diseases and also referring to the results of neuroprotection in the SH-SY5Y and HT-22 tests, the compounds were examined for their redox properties in chemical tests.^10,18^ Due to the potential for this chemotype to exhibit antioxidant properties through multiple mechanisms, compounds **WA-22** and **PPK-32** were subjected to the phosphomolybdenum test.

**Figure 13:**
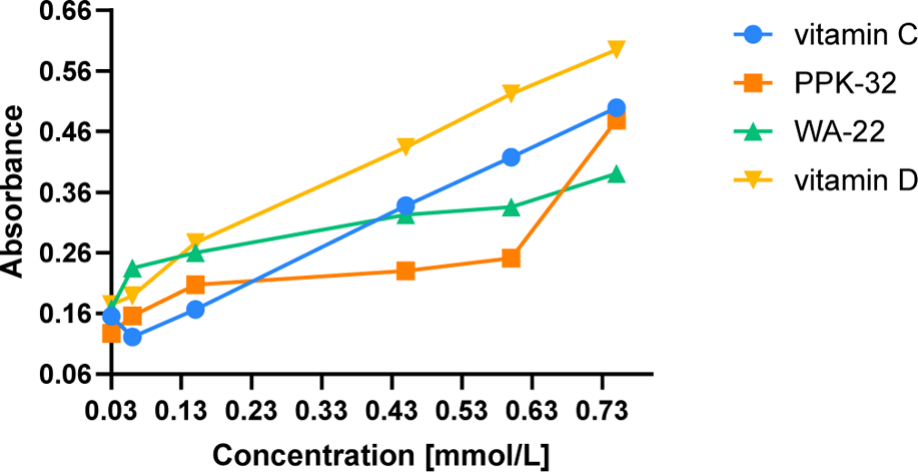
Absorbance vs. concentration [mmol/L] graphs of total antioxidant capacity for **WA-22** and **PPK-32** vs reference vitamins C and D.

Notably, the antioxidant power of the tested compounds was determined based on the reduction of molybdenum ions from Mo(VI) to Mo(V) by the method described by Prieto et al.^18^ As shown in Figure 13, both compounds demonstrated relatively strong antioxidant properties.

In contrast to the reference vitamins, this dependence was not proportional to the concentration increase. In particular, in the range of moderate concentrations for **PPK-32**, the antioxidant activity almost does not increase with the concentration. This stagnation begins at a concentration of ∼100 μM. The weaker antioxidant properties of **PPK-32** in this range were clearly visible, in comparison to both **WA-22** and both reference vitamins (C and D). On the other hand, much more beneficial antioxidant properties of both compounds compared to the reference vitamins can be observed in the range of low concentrations. This behavior seems to be promising from a therapeutical point of view as the compounds demonstrated the 5-HT_6_R affinity at the nanomolar concentration and various neuroprotective actions in the range of 10–50 μM. The activity seen with compound **WA-22** up to a concentration of 0.15 mmol/L surpasses vitamin C used as a reference, and vitamin C exhibited better antioxidant properties only from a concentration of 0.45 mmol/L. Compound **PPK-32**, tested at the lowest concentrations, displayed weaker action than that of its *S*-analogue but distinctly stronger than vitamin C. Intriguingly, a sudden increase of antioxidant action of **PPK-32** to levels comparable to vitamin C, surpassing **WA-22**, was observed at the highest concentrations (>0.65 mmol/L).

### 2.5 In Vivo Studies: Pharmacokinetics

**Figure 14:**
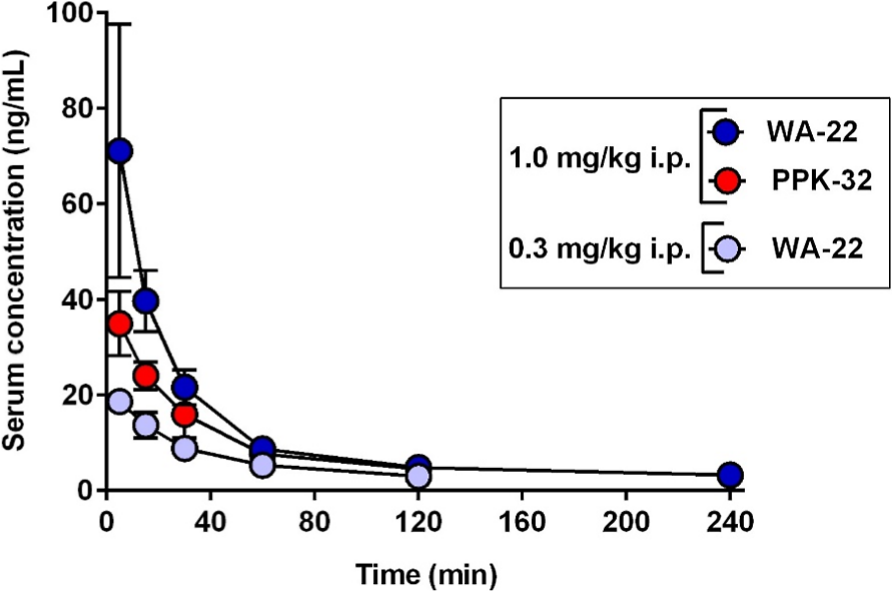
Comparison of the mean (±SD) serum concentration–time profile of **WA-22** (0.3 and 1.0 mg/kg) and **PPK-32** (1.0 mg/kg) in the rat (*N* = 3–4/dose) following i.p. administration. Serum concentration of compound **WA-22**/**PPK-32** (*Y*-axis) was represented using a logarithmic scale. The results of compound **PPK-32** were published.^10^

Serum concentration–time profiles of **WA-22** in rats at doses of 0.3 and 1.0 mg/kg i.p. were compared to the profile of compound **PPK-32**(10) in rats after a dose of 1.0 mg/kg i.p., as shown in Figure 14.

Figure 14 shows that the compound **WA-22** administered at doses of 0.3 and 1.0 mg/kg i.p. was rapidly absorbed from the peritoneal cavity (*T*_max_ = 5.0 min). The peak concentration (*C*_max_) of **WA-22** was 3.8-fold higher after a dose of 1.0 mg/kg i.p. in relation to a dose of 0.3 mg/kg i.p. (*C*_max_ = 71.1 and 18.7 ng/mL, respectively), while the *C*_max_ of compound **PPK-32** was half lower (*C*_max_ = 34.9 ng/mL) than **WA-22** administered at the same dose. The serum concentration was found to be below the lower limit of quantification following 240 min after i.p. administration of **WA-22** and **PPK-32** at doses of 0.3 and 1.0 mg/kg, respectively.

The pharmacokinetic parameters of **WA-22** in rats administered i.p. at doses of 0.3 and 1.0 mg/kg are presented in Table 6.

**Table 6:** Pharmacokinetic Parameters (Mean ± S.D., *N* = 3–4/Group) Calculated Noncompartmental Analysis from the Concentrations of **WA-22** Compound in the Serum after Single Administration at Doses of 0.3 and 1.0 mg/kg^a^

parameters	units	dose	
		0.3 mg/kg i.p	1.0 mg/kg i.p
*C*_max_	ng/mL	18.65 ± 1.87	71.05 ± 26.52
*T*_max_	min	5	5
AUC_0–*t*_	ng·min/mL	840.54 ± 66.81	2537.72 ± 605.18
*V*_*z*_/*F*	L/kg	28.15 ± 6.41	75.43 ± 28.17
CL/*F*	L/h/kg	15.28 ± 0.69	18.77 ± 4.73
*t*_1/2λz_	min	77.20 ± 20.97	168.69 ± 46.71
MRT	min	96.90 ± 24.47	162.08 ± 50.53

a *C*_max_—maximum concentration; *T*_max_—time to reach the maximum concentration; AUC_0–*t*_—area under the serum concentration–time curve from the time of dosing to the time of the last measurable concentration; *V*_*z*_/*F*—volume of distribution at the elimination phase; CL/*F*—oral clearance; *t*_1/2λ*z*_—half-life in the elimination phase; MRT — mean residence time.

The area under the serum concentration–time curve from the time of dosing to the time of the last measurable concentration (AUC_0–*t*_) for the serum was 840.54 ng·min/mL for a dose of 0.3 mg/kg i.p. and 2537.72 ng·min/mL for a dose of 1.0 mg/kg i.p. The apparent volumes of distribution (*V*_*z*_/*F*) during the terminal phase were 28.15 and 75.43 L/kg for doses of 0.3 and 1.0 mg/kg i.p., respectively. The clearance (CL/*F*) was 15.28 L/h/kg for a dose of 0.3 mg/kg i.p. and 18.77 L/h/kg for a dose of 1 mg/kg i.p. The elimination half-life of **WA-22** was 77.20 min for a dose of 0.3 mg/kg i.p. and 168.69 min for a dose of 1.0 mg/kg i.p.

**Figure 15:**
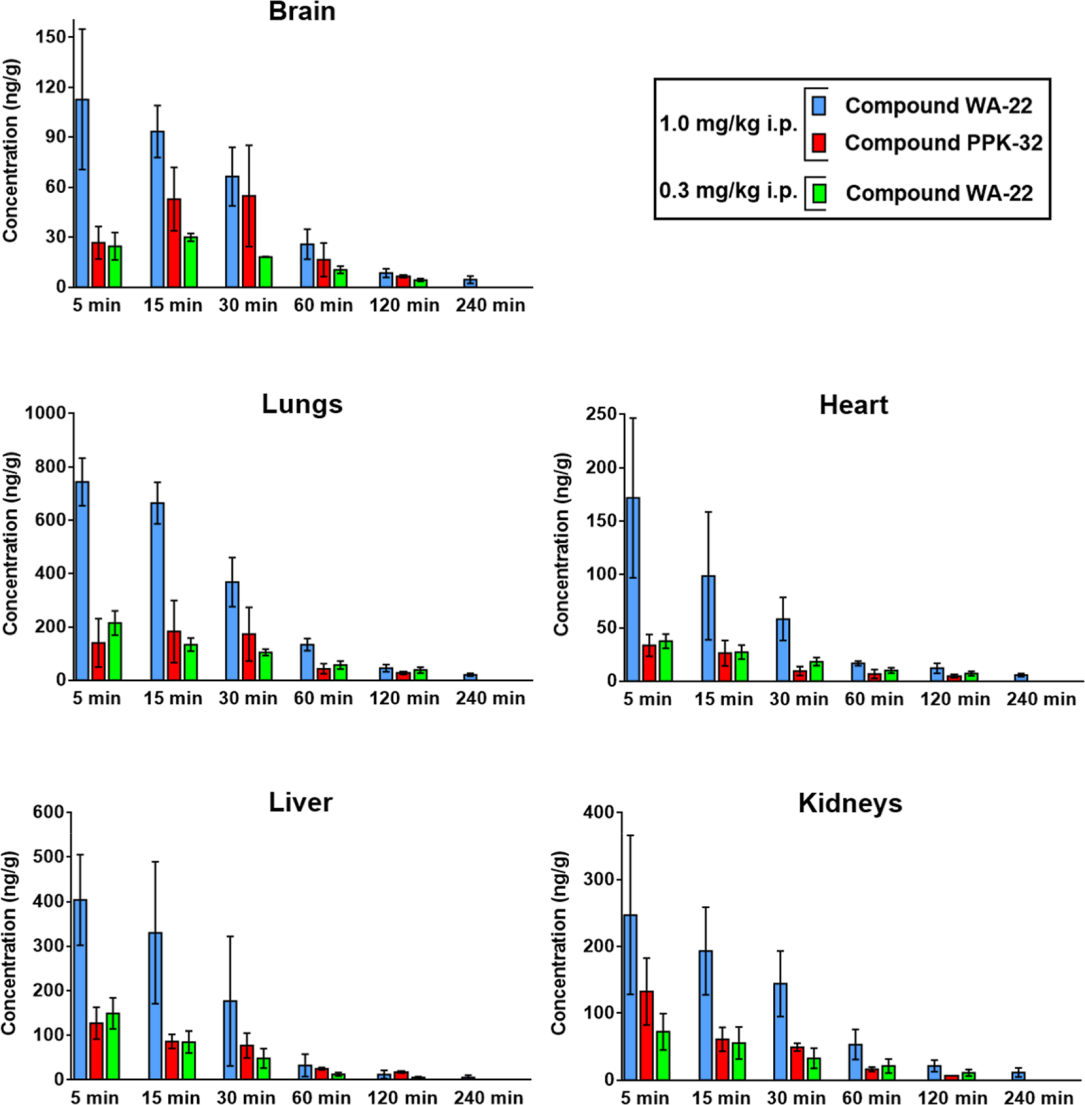
Comparison tissue concentration (mean ± standard deviation (SD)) **WA-22** and **PPK-32** after i.p. administration of **WA-22** at the doses of 0.3 and 1.0 mg/kg and compound **PPK-32** at a dose of 1.0 mg/kg i.p. in rats (*N* = 3–4/dose). The results of compound **PPK-32** were published.^10^

Figure 15 shows a comparison of the mean concentrations of **WA-22** and **PPK-32** in various tissue samples (brain, heart, lungs, liver, and kidneys) at 5, 15, 30, 60, 120, and 240 min after i.p. administration of **WA-22** at doses of 0.3 and 1.0 mg/kg and **PPK-32** at a dose of 1.0 mg/kg i.p. to rats (results were published^10^).

The results showed that **WA-22** was detected in all of the tissues sampled, suggesting that it is well distributed to all of the major organs sampled in this study (Figure 15). The maximum concentrations were observed at the first sampling time point, i.e., 5 min in most tissues examined. **WA-22** was primarily present in organs supplied with abundant blood, such as the lungs, liver, and kidney, indicating that it is mainly metabolized and excreted in those tissues. At 120 min, nearly 80–95% of **WA-22** was cleared in rat tissues and serum, indicating no accumulation of **WA-22**. In the case of the selenium derivative **PPK-32**, concentrations in all tissues were lower at each time point as compared to those of **WA-22** after administration at the same dose (1.0 mg/kg i.p.). The main pharmacokinetic parameters for **WA-22** in rat tissues after doses of 0.3 and 1.0 mg/kg i.p. (*N* = 3–4 rats/group) are summarized in Table 7.

**Table 7:** Mean Values of Pharmacokinetic Parameters Calculated from the Concentrations of **WA-22** in Tissues after Single i.p. Administration at Doses of 0.3 and 1.0 mg/kg (Non-compartmental Analysis Mean ± SD, *N* = 3–4/Time Point)^a^

**WA-22** (i.p.)	organs rat	*C*_max_ (ng/g)	*T*_max_ (min)	*t*_0.5λz_ (min)	MRT (min)	AUC_0–*t*_ (ng·min/g)	AUC ratio
	brain	30.66 ± 3.28	11.67 ± 5.77	48.35 ± 4.46	64.37 ± 3.35	1575.82 ± 182.54	1.89 ± 0.34
	heart	37.51 ± 6.65	5.00 ± 0.00	84.99 ± 19.47	104.04 ± 24.20	1670.68 ± 385.10	2.00 ± 0.52
**0.3**mg/kg	lungs	214.42 ± 45.69	5.00 ± 0.00	105.43 ± 5.42	129.90 ± 11.20	9320.95 ± 1906.43	11.14 ± 2.57
	liver	149.06 ± 34.83	5.00 ± 0.00	48.07 ± 6.68	40.59 ± 5.84	3993.81 ± 1308.63	4.75 ± 1.57
	kidneys	72.35 ± 27.12	5.00 ± 0.00	67.32 ± 12.95	85.27 ± 13.60	3276.54 ± 1455.54	3.93 ± 1.88
	brain	114.79 ± 39.16	8.33 ± 5.77	110.31 ± 24.35	90.57 ± 21.06	5708.89 ± 6488.31	2.46 ± 1.32
	heart	171.75 ± 74.73	5.00 ± 0.00	122.04 ± 18.30	108.54 ± 16.09	6013.14 ± 2336.18	2.63 ± 1.69
**1.0**mg/kg	lungs	743.74 ± 88.91	5.00 ± 0.00	96.48 ± 13.43	72.63 ± 8.12	33528.62 ± 5691.73	14.11 ± 5.62
	liver	403.80 ± 101.31	5.00 ± 0.00	108.96 ± 17.84	51.32 ± 11.56	14056.00 ± 8075.06	6.32 ± 4.96
	kidneys	247.23 ± 119.06	5.00 ± 0.00	121.93 ± 44.52	107.46 ± 36.86	12540.07 ± 4826.76	5.47 ± 3.33

a *C*_max_—maximum concentration; *T*_max_—time to reach the maximum concentration; AUC_0–*t*_—area under the serum concentration–time curve from the time of dosing to the time of the last measurable concentration; *V*_*z*_/*F*—volume of distribution at the elimination phase; CL/*F*—oral clearance; *t*_1/2λz_—half-life in the elimination phase; MRT—mean residence time; AUC ratios—tissue-to-serum AUC_0–*t*_ ratio.

Maximum brain concentration (*C*_max_) after i.p. administration of **WA-22** was reached in the 12th (30.66 ± 3.3 ng/g) and 8th min (114.8 ± 39.2 ng/g) at doses of 0.3 and 1.0 mg/kg, respectively. The AUC_0–*t*_ values estimated for the studied tissues (i.e., brain, heart, lungs, kidneys, and liver) after administration of a dose of 1.0 mg/kg i.p. were on average 3 times higher than those values after the administration of a dose of 0.3 mg/kg i.p. The distribution (AUC ratios) of **WA-22** in all tissues analyzed was higher than unity. The order of AUC ratios was lungs > liver > kidneys > heart > brain (Table 7). The half-lives in the heart and lungs were longer than those in the serum, but elimination half-lives in other tissues were similar to the half-life in the serum (Table 6).

Table 8 shows a comparison of the mean values of the ratio of *C*_max_ and AUC_0**–∞**_ in various tissues to *C*_max_ and AUC_0**–∞**_ in the rat serum after administration of the same dose (1 mg/kg i.p.) of compound **WA-22** and compound **PPK-32**.

**Table 8:** Comparison of Tissue-To-Serum Concentration Ratios (Mean ± SD) Following i.p. Injection at a Dose of 1 mg/kg of **WA-22** or **PPK-32** (*N* = 3–4)^a^

matrix	compound	*C*_max_ (ng/g)/*C*_max_ (ng/mL)	*T*_max_ (h)	AUC_0–∞_ (ng·h/g)/AUC_0–∞_ (ng·h/mL)
serum	**WA-22**		5.00 ± 0.00	
	**PPK-32**		5.00 ± 0.00	
brain	**WA-22**	1.99 ± 1.61	8.33 ± 5.77	2.14 ± 1.18
	**PPK-32**	1.48 ± 0.29	15.00 ± 0.00	1.70 ± 0.35
heart	**WA-22**	2.61 ± 1.09	5.00 ± 0.00	2.09 ± 2.71
	**PPK-32**	0.95 ± 0.13	5.00 ± 0.00	0.81 ± 0.30
lungs	**WA-22**	14.97 ± 7.31	8.33 ± 5.77	13.88 ± 7.95
	**PPK-32**	5.84 ± 4.86	5.00 ± 0.00	7.52 ± 2.77
liver	**WA-22**	6.30 ± 2.69	5.00 ± 0.00	5.11 ± 3.85
	**PPK-32**	3.86 ± 1.80	5.00 ± 0.00	4.20 ± 1.33
kidneys	**WA-22**	3.41 ± 0.59	5.00 ± 0.00	4.96 ± 3.20
	**PPK-32**	4.06 ± 2.30	5.00 ± 0.00	2.34 ± 0.30

a *C*_max_—peak observed concentration; *T*_max_—time of peak concentration; AUC_0–∞_—area under the concentration-time curve from time 0 to infinity.

### 2.6 Behavioral Studies

#### 2.6.1 Effects of 21 Day Administration of **WA-22**, **PPK-32**, and Donepezil on (+)**MK-801**-Induced Memory Disturbances

**Figure 16:**
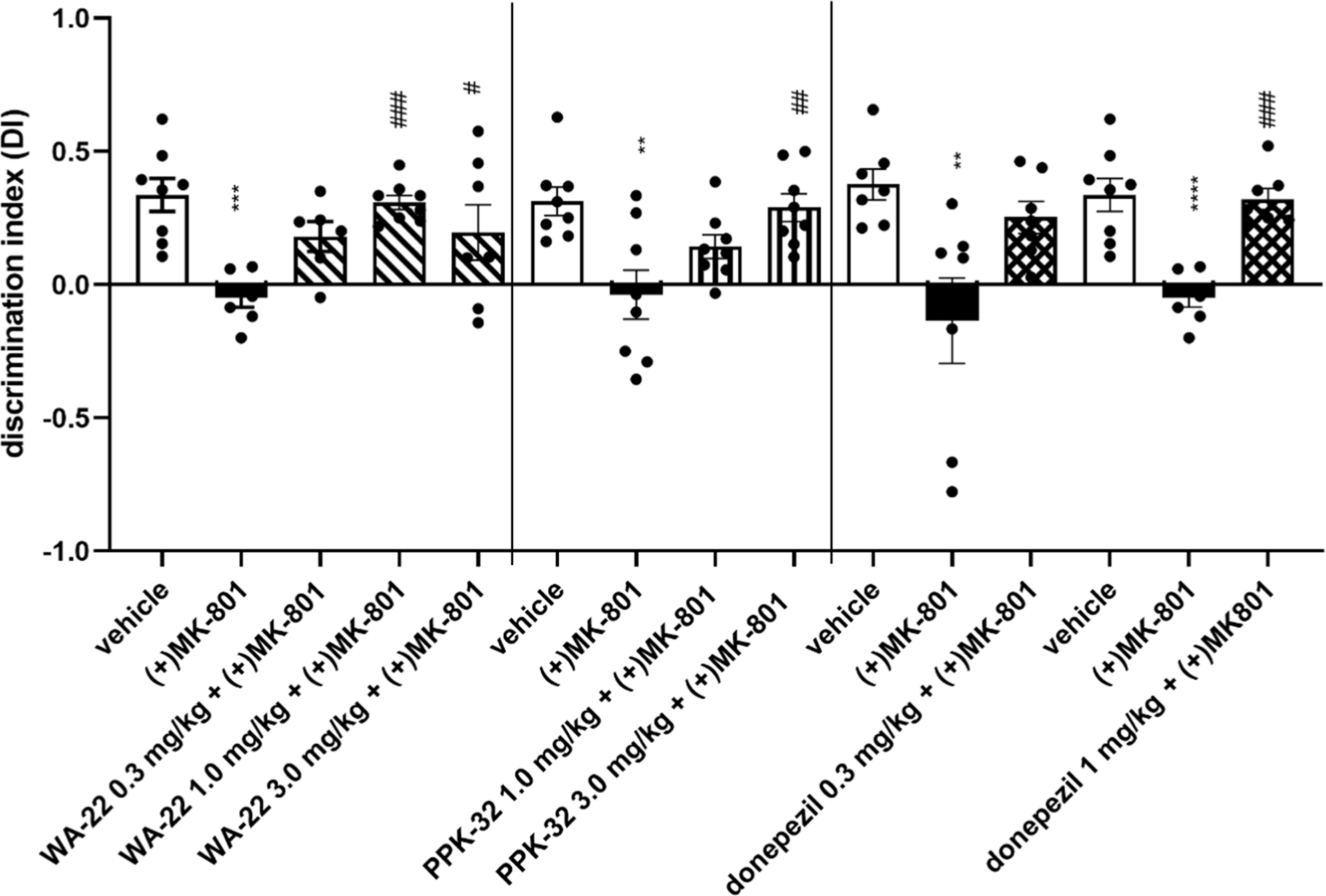
Effects of 21 day administration of **WA-22**, **PPK-32**, and donepezil on **(+) MK-801**-induced memory disturbances. Compounds **WA-22**, **PPK-32**, and **donepezil** were given i.p. once a day for 21 consecutive days, with the last injection 24 h before NORT, **(+)MK-801** was administered i.p. only once, 30 min before the test. Values represent the mean ± SEM of the discrimination index (DI) one-way ANOVA followed by Bonferroni’s posthoc test; **p < 0.001, ***p < 0.0001, ****p < 0.00001 compared to the respective vehicle group; #*p* < 0.05, ##*p* < 0.001, ###*p* < 0.0001 compared to the respective **(+)MK-801**-treated group. *N* = 6–8.

Previously obtained results for compounds **WA-22**(11) and **PPK-32**(10) after their acute administration to rats encouraged us to investigate their ability to reverse **(+)MK-801**-induced memory impairments after chronic administration (21 days) to rats in NORT. The discrimination index (DI) was used to reflect the preference of rats to explore a novel or familiar object; the higher DI value indicates a stronger preference for the novel object. **(+)MK-801** (0.1 mg/kg) induced deficits in memory functions, decreasing DI in a statistically significant manner. **WA-22** given i.p. for 21 consecutive days at a dose of 1 mg/kg/day significantly reversed **(+)MK-801**-induced memory disturbances, measured by the DI level. This activity of compound **WA-22** was also observed for a dose of 3 mg/kg, but memory restoration was not at the same level as that of control rats treated only with the vehicle (Figure 16). Comparing the present results with the acute administration of **WA-22**, which showed statistically significant activity in NORT in the full investigated range of doses (0.3–3 mg/kg),^11^ we observed a lower ability of this compound to reverse memory impairment. However, it should be noted that the ability of **WA-22** to restore memory impairments after chronic administration was still observed and comparable in effectiveness to donepezil administered chronically at the same dose, i.e., 1 mg/kg.

The compound **PPK-32** significantly reversed **(+)MK-801**-induced memory impairment at a chronic dose of 3 mg/kg/day, while a dose of 1 mg/kg/day was inactive in NORT (Figure 16). Furthermore, the activity of **PPK-32** was weaker than that observed after acute i.p. administration to rats.^10^

The reference memory enhancer, donepezil, was administered chronically to reverse memory disturbances at a dose of 1 mg/kg/day, comparable to the activity observed after its acute administration^11^ in NORT (Figure 16).

To avoid biases regarding the effect of treatment on behavioral parameters that may influence the results obtained in NORT, we measured the total exploratory time of objects in the recognition phase (T2). No drug treatment changed the total exploratory time in a statistically significant manner measured during the T2 trial, except for **WA-22** administered at doses of 0.3 and 3 mg/kg/day, where a statistically significant decrease in exploratory activity was observed when compared to **(+)MK-801**-treated rats (Table 9).

**Table 9:** Effect of 21 Days Administration of **WA-22**, **PPK-32**, and Donepezil on **(+)MK-801**-Locomotor Activity in NORT^a^

treatment	dose (mg/kg)	total exploratory time in T2 session (s) (time in interaction with familiar and novel object during 3 s observation) ± SEM
**vehicle + vehicle**	0 + 0	40.38 ± 3.60
**(+)MK-801 + vehicle**	0.1 + 0	47.29 ± 3.63
**WA-22 + (+)MK-801**	0.3 + 0.1	29.00 ± 3.12; *p* < 0.05 vs MK
	1.0 + 0.1	43.62 ± 4.26
	3.0 + 0.1	25.71 ± 2.46; *p* < 0.01 vs MKF(4.31) = 6.4377; *p* < 0.001
**vehicle + vehicle**	0 + 0	31.25 ± 2.89
**(+)MK-801 + vehicle**	0.1 + 0	33.38 ± 2.32
**PPK-32 + (+)MK-801**	1.0 + 0.1	33.25 ± 1.83
	3.0 + 0.1	28.75 ± 2.25 F(3.28) = 0.8477; NS
**vehicle + vehicle**	0 + 0	33.43 ± 3.90
**(+)MK-801 + vehicle**	0.1 + 0	39.43 ± 3.40
**donepezil + (+)MK-801**	0.3 + 0.1	35.14 ± 3.62 F(2,18) = 0.6502; NS
**vehicle + vehicle**	0 + 0	40.38 ± 3.60
**(+)MK-801 + vehicle**	0.1 + 0	47.29 ± 3.63
**donepezil + (+)MK-801**	1.0 + 0.1	40.54 ± 3.20 F(2.19) = 1.1727; NS

a Compounds **WA-22, PPK-32,** and **donepezil** were given i.p. once a day for 21 consecutive days, with the last injection 24 h before NORT, **(+)MK-801** was administered i.p. only once, 30 min before the test. Values represent the mean ± SEM of the total exploratory time of both objects during the 3 min test session (T2) compared to the respective vehicle group (one-way ANOVA followed by the Bonferroni’s posthoc test); NS = nonsignificant. *N* = 6–8.

In accordance with the 3R principles, we decided to develop further pharmacological experiments for the compound that showed a higher and comparable ability with donepezil to reverse **(+)MK-801**-induced memory impairments in NORT. Hence, for the interaction study with donepezil, **WA-22** was chosen.

#### 2.6.2 Influence of 21 Day Administration of WA-22 on Memory in NORT

**Figure 17:**
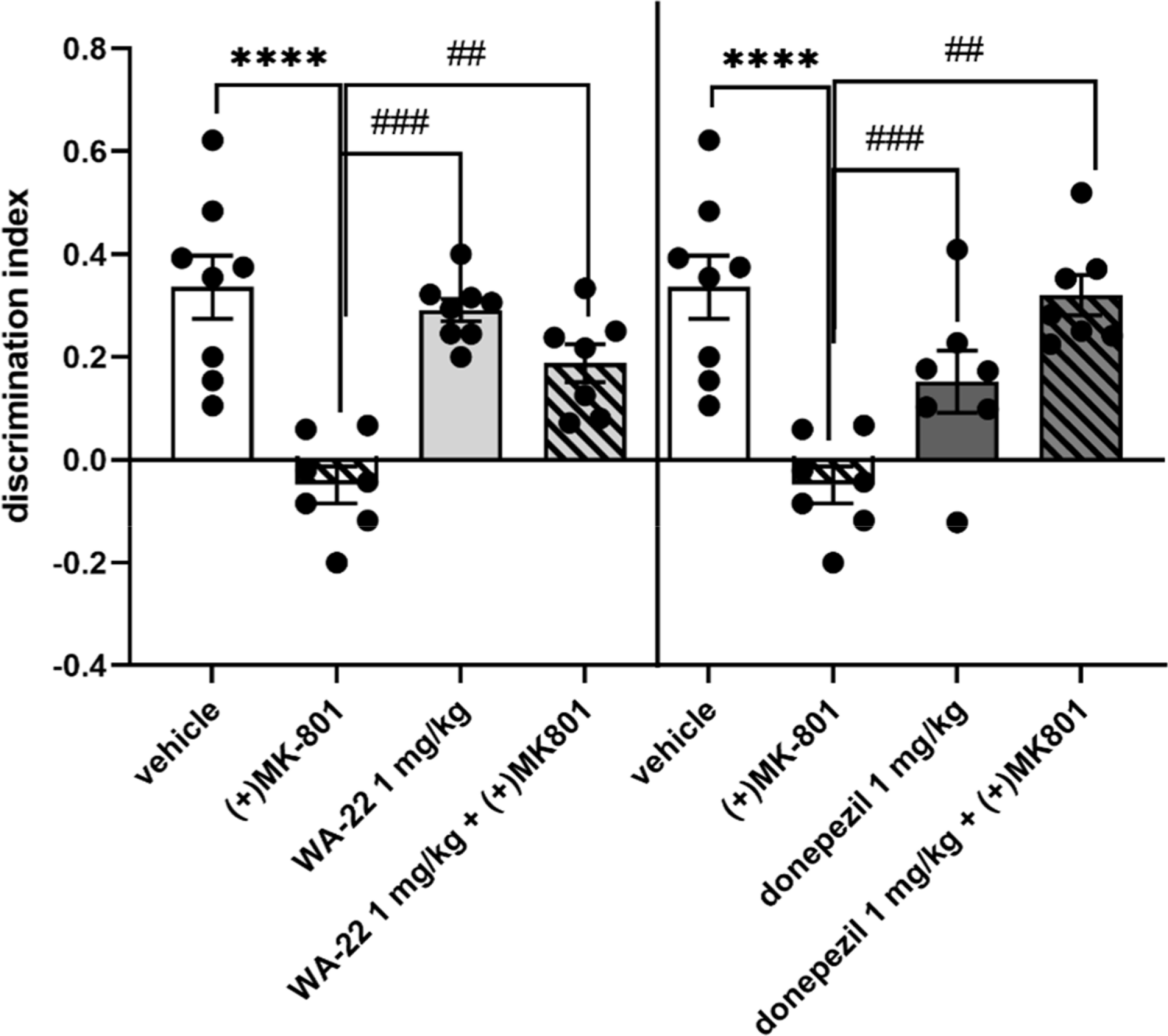
Influence of 21 day administration of **WA-22** and donepezil on rats’ memory in NORT. Compound **WA-22** and donepezil were given i.p. once a day for 21 consecutive days, with the last injection 24 h before NORT, **(+)MK-801** was administered i.p. only once, 30 min before the test. Values represent the mean ± SEM of the discrimination index (DI) one-way ANOVA followed by Bonferroni’s posthoc test; ****p < 0.00001 compared to the respective vehicle group; ##*p* < 0.001, ###*p* < 0.0001 compared to the respective **(+)MK-801** treated group. *N* = 6–8.

To be sure of the specific ability of compound **WA-22** to reverse **(+)MK-801**-induced memory impairments in NORT, the minimal active chronic doses of **WA-22** (1 mg/kg/day) and donepezil (1 mg/kg/day) were chosen and further investigated after their chronic administration to rats. No memory changing was observed after 21 days administration of **WA-22** or donepezil compared to vehicle-treated rats, while the ability of these compounds to reverse **(+)MK-801**-induced memory impairments was sustained (one-way ANOVA for **WA-22**: F(3,27) = 19.132; *p* < 0.00001; and for donepezil: F(3,25) = 11.961; *p* < 0.0001) (Figure 17).

#### 2.6.3 Influence of the 21 Day Joint Administration of **WA-22** and Donepezil on **(+)MK-801**-Induced Memory Deficits in NORT

**Figure 18:**
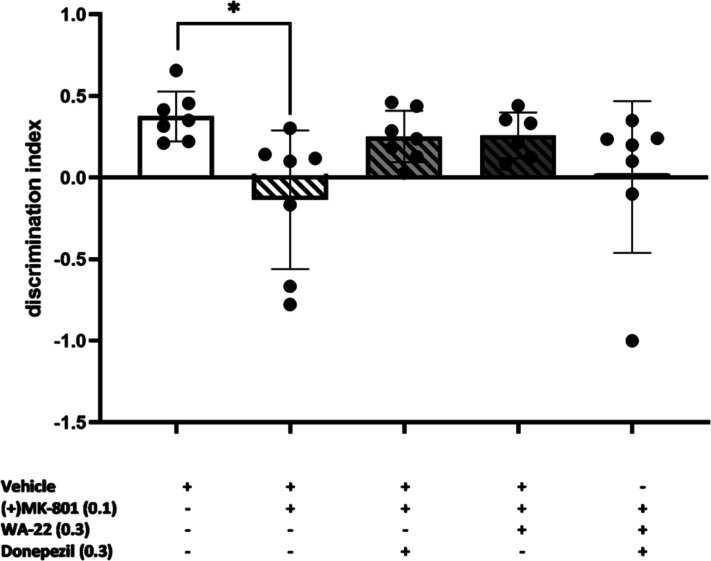
Effect of the 21 day joint administration of **WA-22** and donepezil on **(+)MK-801**-induced memory deficits in NORT. Compound **WA-22** and donepezil were given i.p. once a day for 21 consecutive days, with the last injection 24 h before NORT, **(+)MK-801** was administered i.p. only once, 30 min before the test. Values represent the mean ± SEM of the discrimination index (DI) one-way ANOVA followed by the Bonferroni’s posthoc test; *p < 0.05 compared to the respective vehicle group; *N* = 6–8.

To assess the ability to reverse **(+)MK-801**-induced memory impairments in the interaction experiment, the lower doses of **WA-22** (0.3 mg/kg/day) and donepezil (0.3 mg/kg/day) were investigated jointly in rat NORT. Unfortunately, combined administration of compound **WA-22** and donepezil for 21 consecutive days did not reverse **(+)MK-801**-induced memory deficits; no statistically significant interaction was observed (two-way ANOVA F(1,24) = 0.4615; NS) (Figure 18). In addition, no changes in the exploratory activity were observed (Table 10).

**Table 10:** Effect of 21 Day Administration of **WA-22**, Donepezil, or Coadministration of **WA-22** and Donepezil on Exploration Activity of Rats in NORT^a^

treatment	dose (mg/kg)	total exploratory time in T2 session (s) ± SEM
vehicle + vehicle		33.4 ± 3.9
(+)MK-801 + vehicle	0.1	39.4 ± 3.4
(+)MK-801 + donepezil	0.1 + 0.3	35.1 ± 3.6
(+)MK-801 + WA-22	0.1 + 0.3	25.3 ± 4.5
(+)MK-801 + donepezil + WA-22	0.1 + 0.3 + 0.3	24.7 ± 2.1 F(4, 30) = 1.4150; NS

a Compound **WA-22** and donepezil were given i.p. once a day for 21 consecutive days, with the last injection 24 h before the NOR test; MK-801 was administered i.p. only once, 30 min before the test. Values represent the mean ± SEM of the total exploratory time of both objects during the 3 min test session (T2) compared to the respective vehicle group (one-way ANOVA followed by the Bonferroni’s posthoc test); NS = nonsignificant. *N* = 6–8.

## 3 Discussion

The studies performed gave comprehensive chemical and biological results in order to estimate the neurobiological profile of two chalcogen-differing analogues, the thioether **WA-22** and the Se-ether **PPK-32**, in search of a valuable innovative drug candidate to be used for a more effective therapy of AD.

Promising in vitro results for **WA-22** enabled in vivo tests to be carried out regarding its potential procognitive effect in rat NORT. Single administration of **WA-22** at doses of 0.3–10 mg/kg^11^ and chronic treatment at a dose of 1 mg/kg (this study) did not show a beneficial effect on memory improvement. But at the same time, **WA-22** did not disturb the recognition memory of tested animals. Similarly, donepezil (1 mg/kg), administered acutely^11^ and repeatedly (this study), did not improve rats’ memory but also did not disturb it, as reflected in the values of the calculated DI. The studies published so far on the effect of **WA-22** administered acutely on memory deficits caused by **(+)MK-801** showed statistically significant differences in DI values. Compound **WA-22** at all tested doses, i.e., 0.3 mg/kg, 1 mg/kg, and 3 mg/kg, showed a significant and specific effect on memory impairments compared to a group treated with **(+)MK-801** alone. Similarly, donepezil at a dose of 1 mg/kg improved recognition memory disturbed by **(+)MK-801**.^11^ Based on the above results, the doses of **WA-22** (0.3, 1, and 3 mg/kg) and donepezil (1 mg/kg) were selected for chronic studies. Repeated administration of **WA-22** reversed **(+)MK-801**-induced memory deficits comparable with the activity of the reference donepezil administered at the same schedule.

It seems that the most advanced research is currently being carried out on the participation of 5-HT_6_R ligands in cognitive processes, where such 5-HT_6_R antagonists as idalopirdine or intepirdine were included in clinical trials.^19−21^ Unfortunately, those studies failed due to the lack of significant efficacy of these substances used in combination with donepezil. However, another antagonist of 5-HT_6_R, compound **SUVN-502**, is currently in clinical trials in patients with mild AD as an add-on therapy in combination with donepezil and memantine.^19,22^

As results of pharmacokinetics studies, this first direct comparison of **WA-22** and **PPK-32** shows that at the dose studied (1 mg/kg), the distribution pharmacokinetics in the serum and tissue differs following a single i.p. administration of both compounds to Wistar rats. Both studied compounds administered at 1 mg/kg i.p. were rapidly absorbed from the rat peritoneal cavity (*T*_max_ = 5.0 min), indicating their quick absorption into the blood. Peak serum concentration of **WA-22** was 2 times higher (*C*_max_ = 71.1 ng/mL) when compared to that of **PPK-32** (*C*_max_ = 34.9 ng/mL ^10^). Similarly, the area under the concentration–time curve from the time of dosing to infinity (AUC_0–∞_) for **WA-22** in the serum was 3352.87 ng·min/mL and was 1.8 times higher than for **PPK-32** (AUC_0–∞=_1833.92 ng·min/mL; *p* < 0.01 ^10^), indicating better absorption from the peritoneal cavity of **WA-22** than **PPK-32**. Moreover, the apparent volume of distribution (*V*_*z*_/*F*) in the terminal phase was not significantly larger for **WA-22** (*V*_*z*_/*F* = 75.4 L/kg) than for **PPK-32** (*V*_*z*_/*F* = 54.8 L/kg; *p* > 0.05); however, these values were approximately 113-fold and 82-fold higher, respectively, than the average volume of total body water in rats (0.67 L/kg).^23^ These data suggest that both compounds could be extensively distributed to tissues and organs with a high degree of binding. Consequently, CL/*F* (calculated as dose/AUC) in the serum for **WA-22** was almost 2-fold lower compared to **PPK-32** (*p* < 0.001), but as a consequence, both compounds were characterized by a slow terminal elimination, resulting in a favorable value for the serum elimination half-time (*t*_0.5λz_ = 168.7 min for **WA-22** and 68.7 min for **PPK-32**; *p* < 0.01 ^10^).

Distribution, the most important area of pharmacokinetic research for central activity, was assessed in the brain, heart, liver, lungs, and kidney homogenates prepared at 5, 15, 30, 60, 120, and 240 min after a single i.p. 1 mg/kg dose of **WA-22.** The tissue distribution profile of **WA-22** was assessed over time (Figure 14), revealing that this compound was widely distributed in all organs examined. Maximal **WA-22** concentrations were already measured at the first observation time point, i.e., five min, after which they gradually decreased over the following 235 min, indicating that **WA-22** does not accumulate substantially in any of the analyzed sites (Figure 14).

Differences in the tissue distribution might lead to differences in their pharmacological activity.

Comparing the distribution of **WA-22** in the analyzed rat tissues in relation to **PPK-32** (Figure 15 and Table 7), it can be assumed that both compounds were widely distributed in the kidneys, liver, lungs, heart, and brain, explaining the large apparent distribution volume estimated from the serum concentration data. The highest tissue concentrations of **WA-22** and **PPK-32** (*C*_max_ ratio and AUC_0–∞_ ratio, Figure 15 and Table 7) were found in the lungs, followed by the liver and the kidneys, which implied that the distribution of both compounds depends on the blood flow or the perfusion rate of an organ. Moreover, according to the concentration–time profiles examined in the liver and kidneys, **PPK-32** decreased more rapidly in the kidneys (*t*_1/2λz_ = 44 min) than in the liver (*t*_1/2λz_ = 85 min; *p* < 0.01). These results show that the kidneys were probably less exposed to **PPK-32** accumulation compared to the liver. However, in the case of **WA-22**, the elimination in both, the kidneys (*t*_1/2λz_ = 122 min) and the liver (*t*_1/2λz_ = 109 min, *p* > 0.05), was similar (Figure 15 and Table 7). Notably, the concentration of **WA-22** in the brain tissue was high, reaching 114.8 ng/g after 5 min (Figure 15). The serum concentration at this time was lower (71.1 ng/mL). In the case of **PPK-32**, the brain concentration was lower than that of **WA-22** but reached the highest value of 30 min after administration (*C*_max_ = 54.9 ng/g, Figure 15).

Indeed, the brain-to-serum *C*_max_ and AUC ratio exceeded the value of 1 (“good” CNS distribution)^24^ for the same dose and route of administration (Table 7). Brain clearance was faster for **PPK-32** (*t*_0.5λz_ = 58.64 min) than for **WA-22** (*t*_0.5λz_ = 110.31 min), but both compounds maintained high concentrations in the brain 5 min (**WA-22**) and 15 min (**PPK-32**) after administration (Figure 15), suggesting their good bioavailability and metabolic stability. **WA-22** was still detected in all analyzed rat organs at 240 min after administration, while **PPK-32** was detected up to 120 min after administration.

Both pharmacokinetic and behavioral studies performed have confirmed a good blood–brain barrier (BBB) penetration for both compounds, especially for the thioether analogue **WA-22**. This speaks in favor of these compounds in the context of the search for new AD therapy^25^ and in general for CNS-drugs, as insufficient drug delivery into the brain leads to a low therapeutic efficacy as well as aggravated side effects due to the accumulation in other organs and tissues.^25^ Transport routes of drug molecules across the BBB occur via various pathways, predominantly via passive diffusion and active transport with the contribution of a variety of transport proteins appropriate for molecules with specific chemical properties and different sizes.^26−29^ Among them, BBB active drug efflux transporters of the ATP-binding cassette gene family are important determinants of drug distribution to, and elimination from the CNS, with the main member, *P*-glycoprotein (Pgp), transporting a huge variety of lipophilic drugs out of the brain capillary endothelial cells that form the BBB.^26^ Expression of Pgp at the BBB in AD is unchanged compared to age-matched controls.^27^

In the case of compounds **WA-22** and **PPK-32,** the predominant way of their influx blood-to-brain seems to be passive diffusion due to the compounds’ physicochemical properties, i.e. molecular weight (400–500 Da), appropriate hydrophobicity and tendency to be nonionized at pH > 7 (blood pH ∼7.4, brain fluid pH ∼7.2), observed on the basis of our DFT calculations. The last property reduces the probability of their active influx via ion transporters, although the methyl-piperazine-1,3,5-triazine moiety, which seems to be bioisosteric with the 1-methyl-4-phenyl-1,2,3,6-tetrahydropyridine, a known substrate for OCT1/2 (organic cation transporters) that contribute to influx into the brain, suggests that **WA-22** and **PPK-32** may also be captured by this protein. Taking into account some moieties corresponding to thiamine B1 and folic acid B9, the 1,3,5-triazine derivatives (**WA-22** and **PPK-32**) could also be susceptible to active transport via sodium-dependent multivitamin transporter (SMVT) that transports multivitamins from blood-to-brain as well as the vitamin transporter reduced folate carrier-1 (RFC1).^28^ In contrast, the concentration of the triazine 5-HT_6_R agents, **WA-22** and **PPK-32**, in the brain is likely limited by efflux pump action. Pgp (ABCB1) seems to be the most appropriate one as features corresponding to the pharmacophore of the triazine 5-HT_6_R antagonists (e.g., 2 aromatic/hydrophobic moieties) frequently occur among the various models of pharmacophores for Pgp modulators. However, those hypotheses need much wider experimental studies to be revised.

In summary, these results suggest that both compounds exhibited favorable pharmacokinetic properties, similar to those of known drugs in rat models. **WA-22** was rapidly absorbed after i.p. administration, and the brain-to-serum AUC ratio was ∼2.17, indicating a good distribution of **WA-22** in the brain. During the tissue distribution evaluation, **WA-22** was found to be the highest in highly perfused organs, such as the lungs, liver, and kidney. This work lays a theoretical foundation for further pharmacological and toxicological research and has new drug development value in a group of 4-(4-methylpiperazin-1-yl)-6-(2-(naphthalen-2-ylthio)propan-2-yl)-1,3,5-triazin-2-amine derivatives.

The chemical characteristics based on crystallography-supported quantum chemistry calculations allowed us to partially explain the pharmacodynamic and pharmacokinetic differences demonstrated by the tested compounds. Considering the variability of the pH in the environment and the globally understood diversity of chemical conditions in complex pharmacokinetic processes, from administration to reaching the brain, the role of orbital energy differences may be meaningful. Thus, HOMO and LUMO energy differences found between **WA-22** and **PPK-32**, with respect to their various ion-state, may explain the relatively lower stability and, as an effect, lower concentration in the brain (*C*_max_) of the selenium compound **PPK-32** compared to its sulfur analogue **WA-22**. In the chronic administration assay, the stronger reactivity of **PPK-32**, and therefore lower stability, translates into a much weaker effect despite the neuroprotective benefits at the gene level associated with the administration of **PPK-32** compared to **WA-22**. It is worth emphasizing that neither the acute nor the chronic period in the conducted behavioral studies is long enough to demonstrate real neuroprotective effects against the years-long development of the neurodegeneration associated with AD. In this context, the extended in vitro neurobiological screening performed significantly enriched our knowledge of the broader neuroprotective potential of both compounds and, consequently, of their therapeutical possibilities against “multi-mechanistic” neurodegenerative diseases such as AD. Thus, based on the obtained results from this and previous studies, either **WA-22** or **PPK-32** seem to be still promising for innovative AD therapy, but at this stage of preclinical studies, the thioether **WA-22** turned out superior to **PPK-32** due to its better pharmacokinetic profile, which also translated into a significantly better effect in the behavioral tests after chronic administration. However, selenium compound **PPK-32** has shown broader neuroprotective profiles, especially at the genetic level. Its ability to suppress BACE1 and translocate NRF2 to the nucleus gives great hope for inhibiting the progression of AD and counteracting neurodegeneration. Therefore, this selenium compound, which also shows a rather favorable safety profile, seems to be particularly interesting for developing an innovative and effective AD therapy. However, it requires improving the pharmacokinetic properties—in particular, greater stability leading to a longer activity in the brain. Thus, chemical modifications and suitable formulations, including the newest trends (e.g., nanoparticles), might be a good solution for the further development of this chemical class of compounds.

## 4 Materials and Methods

### 4.1 Crystallographic Studies

Single crystals of **WA-22**, suitable for structure determination, were obtained from n-propyl acetate by slow solvent evaporation at room temperature.

Data for single crystals were collected using a XtaLAB Synergy-S diffractometer equipped with a Cu (1.54184 Å) Kα radiation source and graphite monochromator. The phase problem was solved by direct methods using SIR-2014 ^29^, and all non-hydrogen atoms were refined anisotropically using weighted full-matrix least-squares on F^2^. Refinement and further calculations were carried out using SHELXL.^30^ The hydrogen atoms bonded to carbons were included in the structure at idealized positions and were refined using a riding model with *U*_iso_(H) fixed at 1.5 *U*_eq_(C) for the methyl groups and 1.2 *U*_eq_(C) for the other hydrogen atoms. Hydrogen atoms attached to nitrogen atoms were found from the difference Fourier map and refined without any restraints. For molecular graphics, the MERCURY^31^ program was used.

Crystallographic data for **WA-22**: C_21_H_28_SN_6_^2+^·2Cl̅, *M*_r_ = 467.45, wavelength 1.54184 Å, crystal size = 0.04 × 0.24 × 0.43 mm^3^, monoclinic, space group *P*2_1_/*c*, *a* = 21.4118(2) Å, *b* = 6.4399(4) Å, *c* = 18.4684(1) Å, β = 111.5552(8)°, *V* = 2368.51(3) Å^3^, *Z* = 4, *T* = 100(2) *K*, 73897 reflections collected, 5152 unique reflections (*R*_int_ = 0.0673), *R*_1_ = 0.0354, w*R*_2_ = 0.0932 [I > 2σ(I)], *R*_1_ = 0.0363, w*R*_2_ = 0.0940 [all data].

CCDC 2412306 contains the supplementary crystallographic data, which can be obtained free of charge from the Cambridge Crystallographic Data Centre at www.ccdc.cam.ac.uk/data_request/cif.

### 4.2 Computational Methods

Geometry optimization was carried out using the hybrid meta-GGA TPSSh functional^32^ combined with the triple-valence def2-TZVPP basis set^33^ (abbreviated as TZVPP). The TPSSh functional was selected based on the test calculations done previously.^34^ Vibrational frequencies were calculated in the harmonic oscillator approximation in order to confirm local minima and to determine thermal corrections to the Gibbs energy. The geometries of the compounds were optimized for the gas phase and the simulated aqueous solution by applying the PCM.^35^ For comparison purposes, in selected cases, solvent effects were also estimated by single-point calculations on gas phase-optimized structures. The energies of compounds in the gas phase and aqueous solution were corrected for dispersion interactions according to the DFT-D3(BJ) approach.^36,37^

Vertical ionization potential (IP_v_) and vertical electron affinity (EA_v_) were calculated for gas phase compounds as
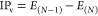

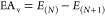
where *E*_(*N*–1)_, *E*_(*N*)_, and *E*_(*N*+1)_ are the total energies of the cationic, neutral, and anionic systems at the neutral geometry. Adiabatic ionization potential (IP_ad_) and adiabatic electron affinity (EA_ad_) were calculated analogously using relaxed geometries of the cationic and anionic systems, respectively.

Electronic chemical potential (μ), absolute electronegativity (χ), chemical hardness (η), and electrophilicity index (ω) were estimated as^38−40^
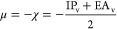

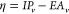

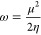


The electronic properties of the systems studied were also analyzed using NPA^41,42^ and the Wiberg bond indexes.^43^

All calculations were done with the Gaussian 16 software.^44^

### 4.3 In Vitro Cytotoxic Effect

This study used the human neuroblastoma cell line SH-SY5Y (ATCC no. CRL-2266) to investigate the effect on the neuronal cells. The cells (8 × 10^3^ cells/100 μL/well) were seeded in transparent 96-well plates (Thermo Scientific, Nunc, no. 161093) in DMEM/F12 (Gibco, no. 11039021) supplemented with 10% FBS (Gibco, no. 10500-064) and cultured overnight. The following day, the treatment was carried out on cells that had reached 20–30% confluence. The medium was aspirated and replaced with 100 μL medium per well-containing dimethylsulfoxide (DMSO 0.1%, control cells, Ctr) or increasing concentration of compound **WA-22** (0.78 × 10^–6^ – 50 × 10^–6^ M, performed as 2-fold serial dilution for dose–response analysis). Treatment with the compounds was performed for 27 h. After incubation, the cell viability was examined using an MTS-based CellTiter96 AQueous One Solution Cell Proliferation Assay (Promega, Madison, WI, USA) following the manufacturer’s protocol. Briefly, 20 μL of MTS solution was pipetted into each well containing 100 μL of culture or culture medium (negative control) and incubated at 37 °C for 1 h. The absorbance was measured at 490 nm using a Tecan Spark’s multimode plate reader (Tecan, Männedorf, Switzerland). A reference wavelength of 630 nm was used to subtract the background. IC_50_ values were calculated by fitting a nonlinear regression to a sigmoidal dose–response curve in GraphPad Prism version 8.0.1.

### 4.4 ROS Assay

The ROS measurement was assayed by 2′,7′-dichlorofluorescin diacetate (2′, 7′DCFH_2_-DA, Sigma, no. D6883). The protocol was previously described in detail.^10^ Briefly, SH-SY5Y cells (2 × 10^4^ cells/well/100 μL) were seeded in a black-sided clear-bottom 96-well plate (Thermo Scientific Nunclon Delta Surface no. 137101, Denmark) in DMEM/F12 supplemented with 10% FBS and cultured for 24 h. The following day, all treatments were carried out with warmed HBSS containing 25 mM HEPES (hereafter referred to as HBSS), and during the operational steps, the cells were kept at 37 °C to minimize temperature stress. First, the medium was removed, and the cells were washed once with HBSS and stained with 2′, 7′DCFH_2_-DA (50 μM, freshly prepared in warm HBSS) for 45 min. Next, the cells were pretreated with **WA-22** (10 μM) for 1 h. After that, ROT (32.5 μM) was added for 3 h. Finally, the fluorescence was measured at *E*_*x*_/*E*_m_ = 505/550 nm using the multimode plate reader Tecan Spark.

### 4.5 Cell Culture and Treatments

SHSY5Y cells were grown in DMEM supplemented by 10% FBS, l-glutamine (2 mM), and penicillin (100u/mL)/streptomycin (100 μg/mL). The cell line was tested for mycoplasma using DAPI staining and the LookOut Mycoplasma PCR Detection Kit (MP0035, Merck). The cell line was authenticated after thawing by a morphology check, cell proliferation rate evaluation, and species verification by PCR. Bacteria contamination was excluded. **PPK-32** and **WA-22** treatments were performed at a concentration of 10 and 50 μM for 24 h SHSY5Y cells.

### 4.6 MTS Assay

SHSY5Y cells were trypsinized, harvested, and seeded onto 96-well flat-bottomed plates at a density of 2000 cells/well, then incubated at 37 °C for 72 h in DMEM supplemented with 10% FBS, and treated with **PPK-32**, **WA-22**, or DMSO. Subsequently, cells were subjected to a CellTiter 96 AQueous One Solution Cell Proliferation Assay (Promega), according to the manufacturer’s protocol. The absorbance at 490 nm was evaluated to estimate the cell number.

### 4.7 RNA Extraction, Reverse Transcription, and Real-Time PCR

Total RNA was extracted by a ReliaPrep RNA Tissue Miniprep System (Promega, USA) and reverse transcribed with an iScriptTM cDNA Synthesis Kit (Bio-Rad Laboratories Inc., USA). Quantitative polymerase chain reaction (RT-qPCR) analyses were performed according to the MIQE guidelines. cDNAs were amplified by the qPCR reaction using GoTaq qPCR Master Mix (Promega, Madison, WI, USA). Relative amounts obtained with the 2̂(−ΔCt) method were normalized with respect to the housekeeping gene L32.

Primers:

L32 Forward GGAGCGACTGCTACGGAAG.

L32 Reverse GATACTGTCCAAAAGGCTGGAA.

BACE-1 Forward CCCGGGAGACCGACGAA.

BACE-1 Reverse CACCAGGATGTTGAGCGTCT.

NFkB Forward GCTTAGGAGGGAGAGCCCA.

NFkB Reverse CTTCTGCCATTCTGAAGCCG.

HO-1 Forward ACCTTCCCCAACATTGCCAG.

HO-1 Reverse CAACTCCTCAAAGAGCTGGATG.

NQO-1 Forward GCTGGTTTGAGCGAGTGTTC.

NQO-1 Reverse CTGCCTTCTTACTCCGGAAGG.

SOD-1 Forward AGGCATGTTGGAGACTTGGG.

SOD-1 Reverse TGCTTTTTCATGGACCACCAG.

NRF2 Forward AGGTTGCCCACATTCCCAAA.

NRF2 Reverse ACGTAGCCGAAGAAACCTCA.

### 4.8 Protein Extraction and Western Blot

For total protein extract, cells were lysed in Laemmli buffer, while for nuclear protein isolation, cells were lysed in Lysis Buffer (MgCl_2_ 1.5 mM, KCl 10 mM, Tris-HCl 20 mM pH 7.5, DTT 1 mM) and after 15 strokes with douncer, nuclei, and cytoplasm were separated by centrifugation (1500 RCF, 4 °C, 5 min). Subsequently, the proteins were resolved by SDS PAGE and transferred to 0.45 μm nitrocellulose membrane (162-0115; Bio-Rad Laboratories). The following primary antibodies were used for immunoblotting: α-NRF2 (ab137550, Abcam), α-GAPDH (MAB-374, Millipore Corp.), and α-H3 (06755, Millipore Corp.), the last two used as loading controls (of total and nuclear protein extracts). The immune complexes were detected with horseradish peroxidase-conjugated species-specific secondary antiserum α-rabbit 172-1019 and α-mouse 170-6516 (Bio-Rad Laboratories) and then by enhanced chemiluminescence reaction (Bio-Rad Laboratories). Densitometric protein expression analysis was performed using the Fiji ImageJ image processing package.

### 4.9 Neuroprotection in HT-22

#### 4.9.1 Cell Preparation

A Mouse Hippocampal Neuronal Cell Line (HT-22) was a generous gift from Dr Bartosz Pomierny of the Department of Biochemical Toxicology, Jagiellonian University Medical College, Krakow, Poland. Cells were cultured in Dulbecco’s modified Eagle’s Medium-high glucose (DMEM, GlutaMAX ThermoFisher) supplemented with 10% inactivated fetal bovine serum heat (Thermo Fisher), 100 IU/mL penicillin (Merck), and 100 μg/mL streptomycin (Merck). Cells were cultured in flasks (area 175 cm^2^, Nunc) and incubated at 37 °C, 5% CO_2_. To measure the neuroprotective effect against Aβ1–42, cells were placed in a 96-well culture plate (5 × 10^3^ cells per well, Falcon). To measure the toxicity of tested compounds, cells were placed in a 96-well culture plate (5 × 10^3^ and 2 × 10^4^ cells per well, Falcon). Before the tests, cells were grown for 24 h in the incubator (37 °C, 5% CO_2_).

#### 4.9.2 Preparation of Solutions of Test Compounds

Stock solutions were prepared at a concentration of 10 mM for the tested compounds. A minimum of 1 mg of each tested compound was weighed and dissolved in an appropriate volume of dimethyl sulfoxide (DMSO). Serial dilutions were prepared in DMSO, and then, the diluted compounds were transferred to phosphate-buffered saline (PBS), mixed, and put into a medium with adherent cells. Before the assays, eventual precipitation or opalescence was checked. It is noticed that at 100 μM, many compounds precipitated in PBS.

#### 4.9.3 Preparation of Solution of β-Amyloid (Aβ1–42) Aggregated

Aβ1–42 (Merck) was initially dissolved in 1,1,1,3,3,3- hexafluor-2-propanol (Merck). The solution was incubated at room temperature for 48 h in the dark and mixed on a roller. After this time, the Aβ1–42 solution was evaporated by using nitrogen gas. Next, DMSO was added to obtain a 2 mM stock solution of β-amyloid. The Aβ1–42 was incubated in a medium (DMEM, GlutaMAX ThermoFisher) at 37 °C for 5 days for the aggregation process. This incubation process allowed Aβ1–42 to aggregate, forming the desired molecular structures for further experimentation.

#### 4.9.4 Aggregated aβ1–42 Treatments

On the day of the experiment, the HT-22 cells were treated with the tested compounds at a concentration of 10 and 1 μM. After one h of incubation with the tested compounds, the cells were exposed to aggregated Aβ1–42 at a concentration of 35 μM and incubated at 37 °C for 16 h.

#### 4.9.5 Measurement of Cell Membrane Damage

Cell membrane damage was measured using the bioluminescent ToxiLight bioassay (Lonza), a cytotoxicity highly sensitive assay. After 24 h of treatments, 5 μL of the clear fluid above a sediment was transferred to a 384-well plate (PerkinElmer). Then, 20 μL of adenylate kinase detection reagent was added. The luminescence was measured after 10 min of incubation with a plate reader, POLARstar Omega (BMG Labtech). The results are expressed as a percentage of the control (Aβ1–42), corresponding to the percentage of dead cells concerning the control sample.

#### 4.9.6 Measurement of Reactive Oxygen Species

To detect the ROS, a CellROX reagent (ThermoFisher) was used. After the end of incubation with the tested compounds, the supernatant was removed, and cells were incubated with CellROX (5 μM) in DMEM FluoroBrite (ThermoFisher) for 1 h at 37 °C. The results are expressed as a percentage of control (Aβ1–42). The fluorescence intensity (EX545; EM565 nm) was measured with an ImageXpress Micro XLS (Molecular Devices).

#### 4.9.7 Statistical Analysis

Statistical analysis was performed using GraphPad Prism 8.0. All values are expressed as mean with SD. Differences among groups were evaluated by One-Way ANOVA followed by posthoc analysis (Dunnett’s multiple comparison tests) and were considered statistically significant if *p* < 0.05 (*p < 0.05, **p < 0.01, ***p < 0.001, ****p < 0.0001).

### 4.10 Total Antioxidant Assays

#### 4.10.1 Phosphomolybdenum Method

To 200 μL of each solution (vitamin C and tested compounds; in DMSO), 660 μL of the following solutions were added: 0.6 mol/L sulfuric acid(VI), 4 mmol/L ammonium heptamolybdate, and 28 mmol/L ammonium phosphate. This mixture was incubated at 95 °C for 90 min. Then, the solutions were cooled to room temperature, and their absorbance was measured at 695 nm. A mixture of all reagents and DMSO was used as a control.

### 4.11 Animals

The experiments were performed on male Wistar rats (200–230 g at the beginning of the experiment) *n* = 192 obtained from an accredited animal facility at the Jagiellonian University Medical College, Poland. The animals were housed in groups of four in a controlled environment ambient temperature 21 ± 2 °C; relative humidity 50 ± 10%; 12 h light/dark cycles (lights on at 8:00) in a Makrolon type-3 cage. Standard laboratory food (LSM-B) and filtered water as well as environment enrichment were freely available. Animals were randomly divided into treatment groups using a computer-based random order generator. All of the experiments were performed by two observers unaware of the treatment applied between 9:00 and 14:00 on separate groups of animals.

Rats were injected with the investigated compounds once (in pharmacokinetic studies) and for 21 days (in behavioral studies), and afterward, behavioral tests were conducted and tissue collection was done, as required. For pharmacokinetic studies, animals were fasted prior to dosing by withholding food but not water overnight. After dosing, the food was withheld for an additional 8 h. All animals were used only once. Animals were handled every day and weighed every other day in the chronic part of the experiment. Procedures involving animals and their care were conducted in accordance with current European Community and Polish legislations on animal experimentation. Additionally, all efforts were made to minimize animals’ suffering and to use only the number of animals necessary to produce reliable scientific data. The experimental protocols and procedures described were approved by the I Local Ethics Commission in Cracow (nos 309/2019 and 765/2023) and complied with the European Communities Council Directive of 24 November 24, 1986 (86/609/EEC) and were in accordance with the 1996 NIH Guide for the Care and Use of Laboratory Animals. The research program complies with the commonly accepted ‘3Rs’ (replacement and reduction of animals, refinement of experimental conditions, and procedures to minimize the harm to animals).

### 4.12 Pharmacokinetics

Experimental data for pharmacokinetic studies of **PPK-32** were described previously,^10^ here used only for comparison with those of **WA-22**.

#### 4.12.1 Pharmacokinetic Study Design

To assess the pharmacokinetic profile and tissue penetration of **WA-22,** the male Wistar rats were single i.p. injected with this compound dissolved in tween (vehicle volume 1 mL/kg) at a dose of 0.3 and 1 mg/kg, determined in behavioral studies. The animals were killed by decapitation at 5, 15, 30, 60, and 120 min (for the dose 0.3 mg/kg i.p.) and additionally, 240 min (for the dose 1 mg/kg i.p.) after **WA-22** compound administration (3–4 animals per time point), and blood samples (approximately 5–6 mL) were collected into tubes. Moreover, five tissues (i.e., brain, heart, lungs, kidneys, and liver) were harvested and rinsed with cold saline. Blood was allowed to clot at room temperature for 15–20 min and then centrifuged (3000 rpm for 10 min). The obtained serum and tissues were stored at −80 °C until analysis.

#### 4.12.2 Instruments

The concentrations of compound **WA-22** in the serum and tissue homogenates were measured by a reverse-phase high-performance liquid chromatography method with ultraviolet detection (HPLC/UV). The HPLC system consisted of a Hitachi–Elite LaChrom pump (L-2130) with an ultraviolet–visible detector (L-2400), a LaChrom L-2300 column oven, and an L-2200 autosampler (VWR, Darmstadt, Germany). Peak areas were integrated automatically using an EZChrome Elite v. 3.3.2 software (VWR, Darmstadt, Germany) chromatographic workstation.

#### 4.12.3 Chromatographic Condition

The chromatographic separation of compound **WA-22** and the internal standard (IS) was achieved on a Supelcosil LC-PCN column 250 × 4.6 mm (Sigma-Aldrich, Germany) with 5 μm particles, protected with the Supelcosil LC-PCN guard column (Sigma-Aldrich, Germany) under isocratic conditions. The mobile phase consisting of methanol −10 mM potassium dihydrogen phosphate buffer (pH 4.6) and acetonitrile (51:40:9, v/v/v) was passed under vacuum through a 0.22 μm filter membrane. HPLC analysis with UV detection at 221 nm was performed at a 1 mL/min flow rate, and the column temperature was 38 °C.

#### 4.12.4 Determination of **WA-22** in Serum and Tissue Homogenates

Before analysis, tissue samples were thawed, weighed (200–250 mg), and placed in 5 mL plastic tubes with four volumes (w/v) of PBS (pH 7.4) and homogenized individually using a MICCRA D-1 homogenizer (ART Prozess & Labortechnik GmbH & Co., Germany). Serum (500 μL) or homogenate samples (500 μL) were mixed with an IS solution ((RS)-4-(4-methylpiperazin-1-yl)-6-(1-phenoxypropyl)-1,3,5-triazin-2-amine), 50 ng/mL in methanol. The samples were alkalized with 50 μL of 4 M sodium hydroxide solution, vortex-mixed, and extracted with 1 mL of ethyl acetate/hexane (30/70, v/v) mixture on a shaker (VXR Vibrax, IKA, Germany) for 20 min. After centrifugation (Eppendorf, Mini Spin Plus, Bionovo, Poland), the organic layers were transferred into new Eppendorf tubes containing 100 μL of methanol and 0.1 M sulfuric acid (10/90, v/v) mixture. Then, the samples were shaken and centrifuged again. Finally, 60–90 μL of each acidic layer was injected into the HPLC system. The retention times of **WA-22** and IS were 12.1 ± 0.09 and 8.3 ± 0.08 min, respectively.

The method was validated according to the procedures and acceptance criteria recommended for bioanalytical method validation for pharmacokinetic studies.^45^ The calibration curve of **WA-22** was constructed by plotting the ratio of the analyte to the IS peak area versus the concentration of analyte was linear in the range of 5–200 ng/mL in the rat serum, 5–250 ng/g in the brain and heart homogenates, 5–500 ng/g in liver and kidney homogenates, and 5–1500 ng/g in the lung homogenate. The interday and intraday precision and accuracy of quality control samples evaluated in the serum and tissue homogenates were within 15%. The lower limit of quantification was 5 ng/mL in the serum and 5 ng/g in tissue homogenates. The mean extraction recoveries of **WA-22** were 82.5–88.9% in the rat serum and tissue homogenates. The mean recovery of IS was 83.33 ± 5.2%.

#### 4.12.5 Pharmacokinetic Data Analysis

The serum concentration–time data were analyzed by a noncompartmental method using the WinNonlin 8.2 nonlinear least-squares regression program (Pharsight Corporation, a Certara Company, Princeton, NJ, USA) to obtain pharmacokinetic parameters. The terminal elimination half-life (*t*_0.5λz_) was calculated to be 0.693/λ_*z*_. The area under the serum concentration–time curve from time zero to time t (AUC_0–t_), where t is the time of the last measurable sample, was calculated according to the linear trapezoidal rule. The AUC from time zero to infinity (AUC_0–∞_) was estimated as AUC_0–*t*_ + *C*_*t*_/λ_*z*_, where *C*_*t*_ was the serum concentration of the last measurable sample. The clearance (CL/*F*) was calculated as D/AUC_0–∞_, and the volume of distribution based on the terminal phase (*V*_*z*_/*F*) was estimated as D/(λ_*z*_·AUC_0–∞_), where *F* is the bioavailability of intraperitoneal administration. The MRT is calculated to be AUC_0–∞_/AUMC_0–∞_, where AMUC_0–∞_ is the area under the first moment curve from the time of dosing to infinity. The peak serum concentration (*C*_max_) and the time to reach *C*_max_ (*T*_max_) were read directly from the observations. The AUC ratios were calculated by dividing the AUC_last_ of **WA-22** in the tissue samples by the serum AUC_last_ values of **WA-22**. Data were presented as mean ± SD.

### 4.13 Behavioral Studies in Rats

#### 4.13.1 Drugs

In the NOR test (NORT), rats were randomly divided into experimental groups (*n* = 8/group). In experiments, we investigated ligands of 5-HT_6_R **WA-22** and **PPK-32** suspended in 1% solution of Tween 80 (Sigma-Aldrich, UK), while **(+)MK-801** (hydrogen maleate, Sigma-Aldrich, UK) and donepezil (Donepezil hydrochloride, Sigma-Aldrich, PL) were dissolved in distilled water. All compounds were prepared immediately before administration and were injected intraperitoneally (i.p.) in a constant volume of 2 mL/kg. **WA-22** (at the doses of 3 mg/kg, 1 mg/kg, and 0.3 mg/kg), **PPK-32** (at the doses of 3 mg/kg and 1 mg/kg), and donepezil (at the doses of 1 mg/kg and 0.3 mg/kg) were administered once a day at a constant hour (8 a.m.) during consecutive 21 days, with the last injection 24 h before the test. The control group received 1% Tween 80 on the same schedule. **(+)MK-801** at a dose of 0.1 mg/kg was administered only once, 30 min before the NORT.

#### 4.13.2 NORT

The test was carried out according to the method described by Ennaceur.^46^ NORT was conducted in two black-painted wood boxes (60 cm × 60 cm × 60 cm). A video camera mounted above the boxes recorded all experiments. Two days before the test, the rats were placed in the empty experimental boxes for 5 min as a habituation to the test area. On the third day, the test was performed in 2 trials lasting for 3 min at one-hour intervals. First session, familiarization (T1) with two identical objects (A1 and A2) located in the opposite corners of the boxes, approximately 10 cm from the walls, and second trial, recognition (T2) with one familiar (A) and one novel object (B). Metal cans and glass jars with sand were used as the objects. The boxes were cleaned and dried after each measurement. The exploration time (E) of the objects (such as looking, licking, sniffing, or touching while sniffing) during T2 was measured, and the discrimination index (DI) was calculated according to the following formula: DI = (EB – EA)/(EA + EB). Exploration time shorter than 5 s was eliminated from the study. **(+)MK-801**, used to attenuate learning, was administered at a dose of 0.1 mg/kg (i.p.) 30 min before familiarization phase (T1).

The total exploration time in T2 was used to express the influence of the treatment on the exploratory activities of the animals.

#### 4.13.3 Statistical analysis

Statistical analysis was performed using Statistica 13.3 (TIBCO Software Inc., Palo Alto, CA, USA) software. The normality of data distribution and the homogeneity of variance were checked using the Shapiro–Wilk’s test and Levene’s test, respectively. All behavioral results are shown as the means ± SEM. The data were evaluated by an ANOVA: one-way ANOVA or two-way ANOVA (used in interaction studies when **WA-22** and donepezil were injected jointly for 21 consecutive days), followed by the Bonferroni’s multiple comparison test; *p* < 0.05 was considered significant.

The Student’s *t*-test was used to compare differences in the pharmacokinetic parameters obtained after administration of the same dose (1 mg/kg i.p.) of compound **WA-22** and compound **PPK-32**. Differences were considered to be statistically significant at *p* < 0.05. The data are expressed as mean values with SD. Charts were prepared using GraphPad Prism 9 (GraphPad Software Inc., San Diego, CA, USA).

## Supporting Information Available

Calculated structures of **WA-22**, **PPK-32,** and their protonated forms for simulated water solution; relative energies and Gibbs energies of the neutral and single protonated forms of **WA-22** and **PPK-32** in the gas phase and simulated aqueous solution; selected bond lengths (*R*, Å) and Wiberg bond indexes (*P*) for the neutral and protonated forms of **WA-22** and **PPK-32** in simulated aqueous solution as well as TPSSh(PCM)/TZVPP calculations; and NPA charges for **S**, **Se**, and fragments (**A**, **B**, **C**, **D**, **E**) of the neutral and protonated forms of **WA-22** and **PPK-32** in simulated aqueous solution as well as TPSSh(PCM)/TZVPP calculations and cartesian coordinates (Å) of the structures **WA-22** and **PPK-32** (PDF)
