# Supplementary material for: Procognitive Potential of Neuroprotective Triazine 5-HT6 Receptor Antagonists Tested on Chronic Activity In Vivo in Rats: Computer-Aided Insight into the Role of Chalcogen-Differences on the Pharmacological Profile
Source: ACS Chem Neurosci. 2025 Feb 28;16(6):1190–209. doi: 10.1021/acschemneuro.4c00873 (PMC11926880; doi:10.1021/acschemneuro.4c00873)
Supplement: Supplementary file 1 — cn4c00873_si_001.pdf [file cn4c00873_si_001.pdf]

Supporting Information

Procognitive potential of neuroprotective triazine 5-HT_6 receptor antagonists tested on chronic activity in vivo in rats: Computer-aided insight into the role of chalcogen-differences on the pharmacological profile

Magdalena Jastrzębska-Więsek[1,a,] , Sabrina Garbo[2,a], Agnieszka Cios[1], Natalia Wilczyńska-Zawal[1], Anna Partyka[1], Ewelina Honkisz-Orzechowska[3], Ewa Żesławska[4], Jarosław Handzlik[5], Barbara Mordyl[6], Monika Głuch-Lutwin[6], Alessia Raucci[7], Marius Hittinger[8], Małgorzata Starek[9], Monika Dąbrowska[9], Wojciech Nitek[10], Tadeusz Karcz[3], Alicja Skórkowska[11], Joanna Gdula-Argasińska[6], Kinga Czarnota-Łydka[3], Patryk Pyka[3], Ewa Szymańska[3], Katarzyna Kucwaj-Brysz[3], Clemens Zwergel[7,8,12], Anna Wesołowska[1], Cecilia Battistelli[2]*, Jadwiga Handzlik [3]*

[1]*Department of Clinical Pharmacy, Jagiellonian University, Medical College, 9 Medyczna Street, 30-688 Kraków, Poland; m.jastrzebska-wiesek@uj.edu.pl (M.J-W.); natalia.wilczynska@uj.edu.pl (N.W-Z.); annairena.partyka@uj.edu.pl (A.P.); agnieszka.cios@uj.edu.pl (A.C.); a.wesolowska@uj.edu.pl (A.W.)*

[2]*Department of Molecular Medicine, Sapienza University of Rome, Viale Regina Elena 324, 00161 Rome, Italy; sabrina.garbo@uniroma1.it (S.G.); cecilia.battistelli@uniroma1.it (C.B.)*

[3]*Department of Technology and Biotechnology of Drugs, Jagiellonian University, Medical College, 9 Medyczna Street, 30-688 Kraków, Poland; j.handzlik@uj.edu.pl (J.H.); patryk98.pyka@doctoral.uj.edu.pl (P.P); ewelina.honkisz@uj.edu.pl (E.H-O.); t.karcz@uj.edu.pl (T.K); kinga.czarnota-lydka@uj.edu.pl (K. C-Ł.); ewa.szymanska@uj.edu.pl (E.S.); katarzyna.kucwaj@uj.edu.pl (K.K-B.)*

[4] *Institute of Biology and Earth Sciences, University of the National Education Commission, Krakow, Podchorążych 2, 30-084 Kraków, Poland; ewa.zeslawska@uken.krakow.pl (E.Ż.)*

[5]*Cracow University of Technology, Faculty of Chemical Engineering and Technology, ul. Warszawska 24, 31-155 Krakow, Poland; jhandz@pk.edu.pl (J. Ha.)*

[6]*Department of Pharmacobiology, Jagiellonian University, Medical College, 9 Medyczna Street, 30-688 Kraków, Poland; joanna.gdula-argasinska@uj.edu.pl (J.G-A); monika.gluch-lutwin@uj.edu.pl (M.G.-L.); barbara.mordyl@uj.edu.pl (B.M.)*

[7]*Department of Drug Chemistry and Technologies, Sapienza University of Rome, Piazzale Aldo Moro 5, 00185 Rome, Italy; alessia.raucci@uniroma1.it (A.R.); clemens.zwergel@uniroma1.it (C.Z.)*

[8]*Pharmbiotec gGmbH, Nußkopf 39, 66578 Schiffweiler; m.hittinger@pharmbiotec.de (M.H.)*

[9]*Department of Inorganic Chemistry and Pharmaceutical Analytics, Jagiellonian University, Medical College, Medyczna 9, 30-688 Kraków, Poland; m.starek@uj.edu.pl (M.S.); monika.1.dabrowska@uj.edu.pl (M.D.)*

[10]*Faculty of Chemistry, Jagiellonian University, Gronostajowa 2, 30-387 Kraków, Poland; wojciech.nitek@uj.edu.pl (W.N.)*

[11]*Imaging Laboratory, Center for the Development of Therapies for Civilization and Age-Related Diseases, Jagiellonian University Medical College, Medyczna 9, 30-688 Krakow, Poland; alicja.skorkowska@uj.edu.pl (A.S.)*

[12]*Division of Bioorganic Chemistry, School of Pharmacy, Saarland University, Campus B 2.1, D-66123 Saarbrücken, Germany.*

[a] Co-first authors

*Correspondence: j.handzlik@uj.edu.pl (J.H.) Tel.: +48 126205580; cecilia.battistelli@uniroma1.it (C.B.) Tel.: +39 0649918236

Index

Figure S1. Calculated structures of **WA-22**, **PPK-32** and their protonated forms for simulated water solution. S3

Table S1. Relative energies (ΔE, kJ mol^{-1}) and Gibbs energies at T = 298.15 K (ΔG_{298}, kJ mol^{-1}) for the single protonated forms of **WA-22** and **PPK-32** in gas phase and simulated aqueous solution (PCM).S3

Table S2. Relative energies (ΔE, kJ mol^{-1}) and Gibbs energies (ΔG_{298}, kJ mol^{-1}) of the neutral and protonated forms of **WA-22** and **PPK-32** in simulated aqueous solution. TPSSh-D3(PCM)/TZVPP/ /TPSSh(PCM)/TZVPP calculations. S4

Table S3. Selected bond lengths (R, Å) and Wiberg bond indexes (P) for the neutral and protonated forms of **WA-22** and **PPK-32** in simulated aqueous solution. TPSSh(PCM)/TZVPP calculations. S4

Table S4. NPA charges for **S**, **Se** and fragments (**A**, **B**, **C**, **D**, **E**) of the neutral and protonated forms of **WA-22** and **PPK-32** in simulated aqueous solution. TPSSh(PCM)/TZVPP calculations. S5

Cartesian coordinates (Å) of the structures WA-22 and PPK-32 .... S6

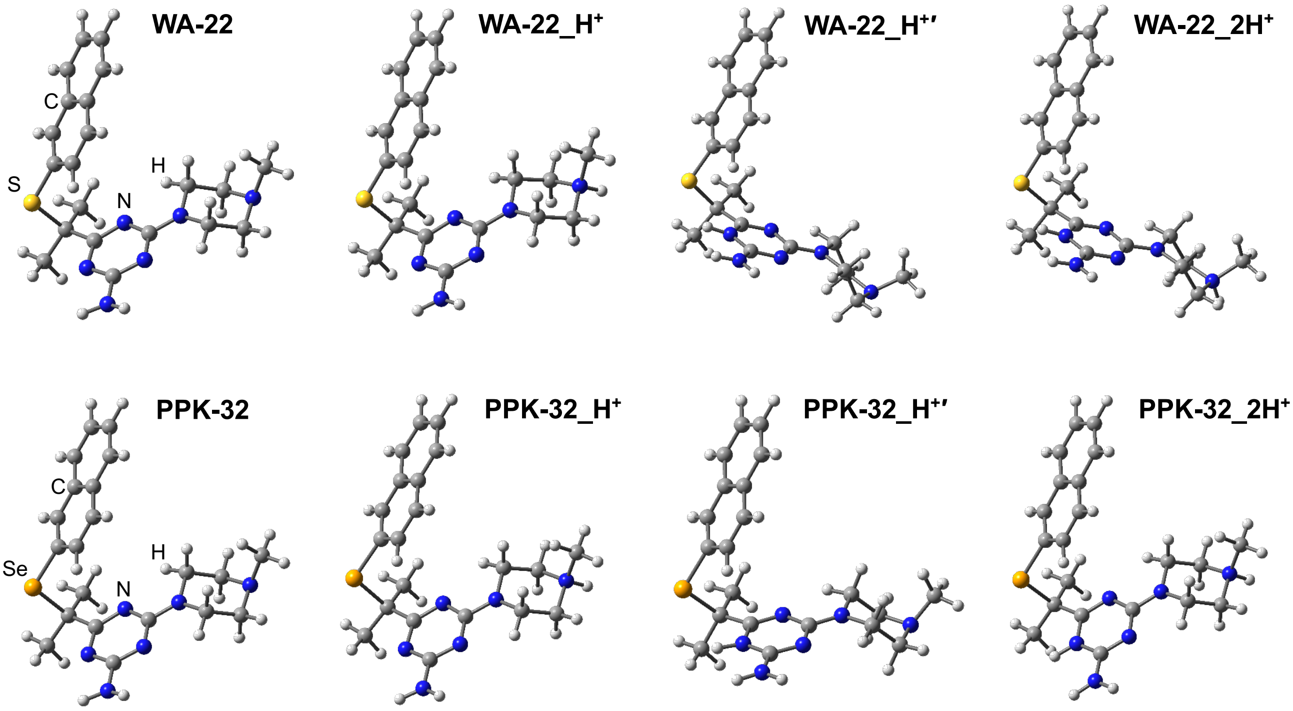

Figure S1. Calculated structures of **WA-22**, **PPK-32** and their protonated forms for simulated water solution.

Table S1. Relative energies (ΔE, kJ mol^{-1}) and Gibbs energies at T = 298.15 K (ΔG_{298}, kJ mol^{-1}) for the single protonated forms of **WA-22** and **PPK-32** in gas phase and simulated aqueous solution (PCM).

method	WA-22_H$^+$ → WA-22_H$^{+\prime}$		PPK-32_H$^+$ → PPK-32_H$^{+\prime}$	
	ΔE	ΔG_{298}	ΔE	ΔG_{298}
TPSSh/TZVPP	-41	-47	-37	-44
TPSSh-D3/TZVPP//TPSSh/TZVPP	-27	-34	-22	-29
TPSSh(PCM)/TZVPP//TPSSh/TZVPP	9	2	11	4
TPSSh-D3(PCM)/TZVPP//TPSSh/TZVPP	22	16	26	19
TPSSh(PCM)/TZVPP	14	7	16	8
TPSSh-D3(PCM)/TZVPP//TPSSh(PCM)/TZVPP	19	11	18	11

Table S2. Relative energies (ΔE, kJ mol^{-1}) and Gibbs energies (ΔG_{298}, kJ mol^{-1}) of the neutral and protonated forms of **WA-22** and **PPK-32** in simulated aqueous solution. TPSSh-D3(PCM)/TZVPP/ /TPSSh(PCM)/TZVPP calculations.

reaction	ΔE	ΔG_{298}
WA-22 + H_3O^+ → WA-22_H$^+$ + H_2O	-172	-162
PPK-32 + H_3O^+ → PPK-32_H$^+$ + H_2O	-172	-162
WA-22_H$^+$ + H_3O^+ → WA-22_2H$^+$ + H_2O	-135	-134
PPK-32_H$^+$ + H_3O^+ → PPK-32_2H$^+$ + H_2O	-140	-135

Table S3. Selected bond lengths (R, Å) and Wiberg bond indexes (P) for the neutral and protonated forms of **WA-22** and **PPK-32** in simulated aqueous solution. TPSSh(PCM)/TZVPP calculations.

species	S/Se-C_{alkyl}		S/Se-$C_{aromatic}$		N-CH_3	
	R	P	R	P	R	P
WA-22	1.878	0.910	1.778	1.037	1.461	1.000
PPK-32	2.033	0.878	1.924	1.009	1.461	1.000
WA-22_H$^+$	1.879	0.908	1.778	1.037	1.500	0.929
PPK-32_H$^+$	2.035	0.875	1.923	1.010	1.500	0.930
WA-22_2H$^+$	1.862	0.936	1.781	1.031	1.502	0.927
PPK-32_2H$^+$	2.036	0.859	1.921	1.013	1.501	0.928

Table S4. NPA charges for **S**, **Se** and fragments (**A**, **B**, **C**, **D**, **E**) of the neutral and protonated forms of **WA-22** and **PPK-32** in simulated aqueous solution. TPSSh(PCM)/TZVPP calculations.

species	*q*(**S**/**Se**)	*q*(**A**)	*q*(**B**)	*q*(**C**)	*q*(**D**)	*q*(**E**)	*q*(**A**+**B**+**C**+**D**)
**WA-22** 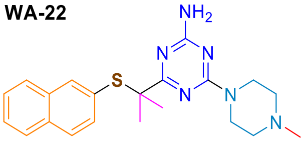	0.19	-0.03	-0.13	-0.05	0.15	-0.12	-0.06
**PPK-32** 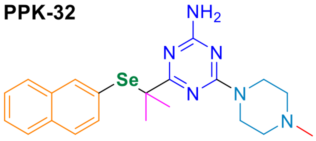	0.25	-0.04	-0.15	-0.05	0.15	-0.17	-0.09
**WA-22_H⁺** 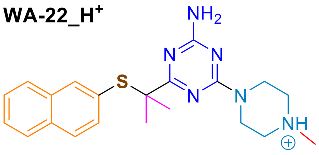	0.19	-0.03	-0.06	0.74	0.29	-0.12	0.93
**PPK-32_H⁺** 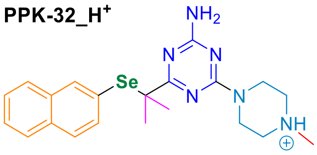	0.26	-0.04	-0.09	0.74	0.29	-0.16	0.90
**WA-22_2H⁺** 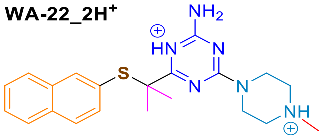	0.22	0.00	0.74	0.85	0.29	-0.09	1.88
**PPK-32_2H⁺** 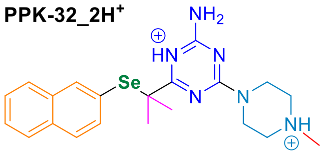	0.31	-0.02	0.70	0.85	0.29	-0.13	1.82

Cartesian coordinates (Å) of the structures WA-22 and PPK-32

WA-22 (gas phase)

H	-2.92486400	1.99668800	2.43160000
H	-1.79318100	3.35426000	2.34609200
C	0.27421100	-2.84440700	1.13671000
S	1.76211600	-2.96748700	-0.00036900
C	0.63154300	-2.07069900	2.40042400
H	1.43696500	-2.58570600	2.92676900
H	-0.24092600	-2.01383600	3.05796300
H	0.94250800	-1.05316800	2.17186700
C	-0.03709300	-4.30939300	1.45840000
H	-0.27009200	-4.86946800	0.55499000
H	-0.90306900	-4.35686500	2.12364300
H	0.81577300	-4.76607300	1.96405100
C	-0.85429400	-2.19312600	0.35439700
N	-1.54358400	-2.96847400	-0.48551900
C	-2.50229000	-2.31716100	-1.16477300
N	-2.79174200	-1.01595600	-1.07668900
C	-2.04130600	-0.34454900	-0.18805200
N	-1.05768400	-0.89591600	0.55947100
N	-3.26324500	-3.06417500	-2.00510000
N	-2.28525300	0.97886700	-0.02014200
C	-3.28638400	1.69355900	-0.80481500
H	-3.88452600	0.96207100	-1.34231200
H	-2.77819300	2.32865600	-1.54231600
C	-4.15533500	2.54512100	0.12973800
H	-4.85231600	3.14660700	-0.45962000
H	-4.74285700	1.87350900	0.76446800
N	-3.39265000	3.43295400	1.00570800
C	-2.39225600	2.66438500	1.74629300
C	-1.47365000	1.81909200	0.85646900
H	-0.83313800	2.46379300	0.24125700
H	-0.83264300	1.17208400	1.44908700
C	-2.85203000	4.60477200	0.32933600
H	-2.11282100	4.39125800	-0.45945200
H	-2.37320600	5.24992100	1.06937800
H	-3.67498900	5.16108800	-0.12523500
C	2.19545200	-1.28554200	-0.37691000
C	1.63533200	-0.64970200	-1.51515700
H	0.90943600	-1.19117100	-2.10862700
C	2.01484700	0.62132500	-1.86159600
H	1.58444400	1.09941100	-2.73529400
C	2.97462100	1.33067200	-1.09743200
C	3.38844700	2.64412400	-1.42701200
H	2.95258000	3.12685200	-2.29524300
C	4.32583800	3.29815500	-0.66523100
H	4.63405900	4.30336300	-0.92822700
C	4.89585500	2.66660700	0.46221000
C	4.51742900	1.39249700	0.80668600
H	4.95415000	0.90343200	1.67072100
C	3.55081500	0.69178600	0.04311400
C	3.14154000	-0.62265000	0.37476300
H	3.58748800	-1.11511700	1.23110800
H	-2.93933800	-3.98997600	-2.22659900
H	-3.85927900	-2.58505900	-2.65766000
H	5.63552700	3.19293800	1.05403300

WA-22 (aqueous solution)

H	-3.16238700	1.81573200	2.40344000
H	-2.14575500	3.26427600	2.35319600
C	0.36858600	-2.75944600	1.16029100
S	1.87118800	-2.86962000	0.03884400
C	0.69257500	-1.93675700	2.40149000
H	1.50935700	-2.40988800	2.94891400
H	-0.18587600	-1.89224200	3.05114500
H	0.97459600	-0.91675600	2.14825500
C	0.10489500	-4.22227400	1.53364000
H	-0.07936100	-4.83196200	0.65097100
H	-0.77608900	-4.27526600	2.17800400
H	0.95879700	-4.62391600	2.08191300
C	-0.77795600	-2.17519200	0.34698600
N	-1.39489200	-2.99425200	-0.50881500
C	-2.38523300	-2.40863200	-1.21078500
N	-2.76372400	-1.12952600	-1.11873900
C	-2.07632600	-0.40620600	-0.21782100
N	-1.06747200	-0.89520100	0.54717200
N	-3.07363000	-3.19850100	-2.06676200
N	-2.40867900	0.89416300	-0.05566900
C	-3.45313000	1.54505200	-0.84428500
H	-3.98530400	0.78498800	-1.40910200
H	-2.98253500	2.23531800	-1.55419100
C	-4.40638600	2.29998200	0.09086100
H	-5.14054200	2.84871700	-0.50344600
H	-4.94361400	1.56816200	0.70294400
N	-3.73618400	3.23587200	0.99626300
C	-2.68171200	2.53909000	1.73664300
C	-1.68441300	1.79064900	0.84417400
H	-1.10084800	2.49746400	0.24264400
H	-0.99611700	1.19904100	1.44065200
C	-3.27459100	4.45474500	0.33514000
H	-2.51096100	4.29940700	-0.44159900
H	-2.85537500	5.12647500	1.08713700
H	-4.12999300	4.94879500	-0.13034700
C	2.29739700	-1.18788000	-0.35045500
C	1.72690900	-0.55522100	-1.48601200
H	0.99570000	-1.09348300	-2.07585600
C	2.10143900	0.71736700	-1.83540200
H	1.66237200	1.19518000	-2.70440000
C	3.06715500	1.42772000	-1.07810400
C	3.47577200	2.74218100	-1.41329400
H	3.03115400	3.22382800	-2.27719000
C	4.41876600	3.39729400	-0.65804000
H	4.72325000	4.40289200	-0.92302000
C	4.99826900	2.76745000	0.46685900
C	4.62422100	1.49289100	0.81733800
H	5.06648100	1.00607400	1.67954900
C	3.65261600	0.79104700	0.05962500
C	3.24663600	-0.52347900	0.39712600
H	3.69686100	-1.00945800	1.25481400
H	-2.70162600	-4.10759800	-2.28465400
H	-3.70281300	-2.76837900	-2.72376200
H	5.74077400	3.29634700	1.05249000

WA-22_H$^+$ (gas phase)

H	1.61986300	-2.63393100	-2.51825100
H	3.02441600	-1.63786500	-2.04956400
C	-2.87081900	1.28378500	-0.97270800

S	-2.34703600	2.54107300	0.32267100
C	-2.17286400	1.52942200	-2.30637900
H	-2.41711900	2.52925100	-2.66643800
H	-2.52016500	0.79921900	-3.04400900
H	-1.09264200	1.43211800	-2.21815200
C	-4.37798700	1.52275500	-1.08827800
H	-4.87946500	1.38097600	-0.13328800
H	-4.80314000	0.81824900	-1.80680100
H	-4.56084200	2.53675300	-1.44665700
C	-2.54542200	-0.07525200	-0.39388000
N	-3.47079200	-0.69503600	0.32950900
C	-3.09147600	-1.87369900	0.85256600
N	-1.87092100	-2.43119000	0.72026600
C	-1.04551400	-1.71281600	-0.03347200
N	-1.31403000	-0.55308900	-0.63421700
N	-4.00592700	-2.54693400	1.56902600
N	0.22808900	-2.22675400	-0.23875700
C	0.70602000	-3.33662400	0.55567200
H	-0.15087000	-3.92550100	0.87983500
H	1.22600500	-2.99304100	1.46115000
C	1.61188900	-4.21342300	-0.29172200
H	2.07548200	-5.01048200	0.28912000
H	1.05685300	-4.64026200	-1.12696600
N	2.74144900	-3.40201600	-0.91391400
C	2.17471500	-2.21153000	-1.68050400
C	1.26454500	-1.38058700	-0.79704100
H	1.83298400	-0.86552000	-0.01184600
H	0.78849300	-0.61042600	-1.40151600
C	3.79800700	-3.00775000	0.07623900
H	3.36166500	-2.35076600	0.82177100
H	4.59166200	-2.48904500	-0.45727800
H	4.18263600	-3.91003300	0.54713300
C	-0.60729300	2.28788600	0.58484400
C	-0.18751900	1.63271700	1.77219600
H	-0.94241700	1.26987000	2.45875400
C	1.14642100	1.48404600	2.05620500
H	1.45626200	1.00599900	2.97994000
C	2.13925200	1.96322800	1.16495200
C	3.52568400	1.81964200	1.42077500
H	3.84078400	1.33675900	2.34015800
C	4.45823400	2.30805600	0.53719200
H	5.51568900	2.20719500	0.75198500
C	4.04680100	2.96859200	-0.64231600
C	2.71067000	3.13003300	-0.91551000
H	2.39269900	3.65085500	-1.81202400
C	1.72280000	2.63266100	-0.02787800
C	0.33872800	2.79698700	-0.27958900
H	0.02661100	3.34486100	-1.16061400
H	-4.91395400	-2.13937100	1.71128900
H	3.19303500	-4.00712300	-1.60072900
H	-3.76830600	-3.42172200	2.00178600
H	4.79205600	3.36216900	-1.32310000

WA-22_H$^+$ (aqueous solution)

H	-2.97989500	1.98857500	2.43678600
H	-1.82441900	3.33467200	2.30515400
C	0.34948300	-2.84398000	1.13079600
S	1.82230600	-2.93029600	-0.03207200
C	0.70476400	-2.05627400	2.38618000

H	1.53016100	-2.54829400	2.90278600
H	-0.15915600	-2.02519900	3.05599300
H	0.98738900	-1.03139800	2.15416600
C	0.08468700	-4.31481700	1.46848000
H	-0.12145500	-4.89962900	0.57393700
H	-0.78220700	-4.38126600	2.13027900
H	0.94827000	-4.73510200	1.98648000
C	-0.80551700	-2.22831900	0.35707600
N	-1.47207100	-3.01926200	-0.48323700
C	-2.46587900	-2.40596000	-1.15625100
N	-2.79999600	-1.11086400	-1.04660800
C	-2.06313000	-0.42725800	-0.16638200
N	-1.05394900	-0.93698100	0.56966500
N	-3.19079100	-3.16145500	-2.00127100
N	-2.35650400	0.89232000	0.01562900
C	-3.34417400	1.58217600	-0.79744700
H	-3.95811700	0.84343100	-1.30538100
H	-2.84889900	2.18924500	-1.56290500
C	-4.24430900	2.43521400	0.08042400
H	-4.92523800	3.03895200	-0.51560500
H	-4.81327900	1.81325500	0.76941400
N	-3.43764100	3.38522100	0.94246800
C	-2.41272900	2.61026100	1.74629200
C	-1.53500400	1.75726300	0.84661300
H	-0.88788300	2.38123900	0.22075900
H	-0.89729400	1.13774800	1.47102200
C	-2.84418000	4.52693700	0.17144300
H	-2.12854600	4.14536100	-0.54897200
H	-2.35152400	5.18972100	0.87769900
H	-3.65188100	5.04538200	-0.33787300
C	2.24388400	-1.24171700	-0.39615500
C	1.67313500	-0.59757400	-1.52507700
H	0.94564900	-1.13170600	-2.12331400
C	2.04295300	0.68085600	-1.85771000
H	1.60429000	1.16735000	-2.72206300
C	3.00259500	1.38632600	-1.08830200
C	3.40528800	2.70709700	-1.40541400
H	2.96101100	3.19732500	-2.26463300
C	4.34255500	3.35730800	-0.63893400
H	4.64268000	4.36772900	-0.89015000
C	4.92222100	2.71604500	0.47944100
C	4.55403900	1.43513000	0.81237600
H	4.99668200	0.93945100	1.66929100
C	3.58811900	0.73813700	0.04295000
C	3.18887000	-0.58301400	0.36189800
H	3.64118300	-1.07842500	1.21301400
H	-2.91608500	-4.11279200	-2.17560800
H	-4.08919400	3.79474500	1.61302300
H	-3.88890400	-2.73270000	-2.58426500
H	5.66039700	3.24119700	1.07381600

WA-22_H$^{+\prime}$ (gas phase)

H	4.96154000	0.30960400	-1.96109500
H	4.98862500	2.01351000	-2.42822300
C	-0.41315700	-1.57165800	-1.41218400

S	-1.95630400	-2.27864000	-0.64431800
C	-0.70761800	-0.34329700	-2.26695100
H	-1.43562900	-0.60785600	-3.03357100
H	0.20776100	-0.00185100	-2.75113700
H	-1.10979600	0.47404500	-1.66923700
C	0.13823200	-2.72591200	-2.28035700
H	0.29505200	-3.63568600	-1.69706900
H	1.08845200	-2.42792800	-2.72756300
H	-0.57443400	-2.94440300	-3.07527500
C	0.63251000	-1.27463200	-0.35007300
N	0.60904500	-2.05081000	0.77565100
C	1.57956300	-1.88262100	1.72622700
N	2.53341900	-0.99602800	1.57738900
C	2.50050300	-0.24691400	0.44810000
N	1.55112700	-0.38249500	-0.53998700
N	1.52571800	-2.64083500	2.82746200
N	3.43459300	0.68036100	0.27483900
C	4.52220800	0.90703500	1.23694800
H	4.41069200	0.21157100	2.06301300
H	4.41898800	1.92794700	1.61532400
C	5.87324100	0.72797400	0.51833400
H	6.67997400	0.95952500	1.21606100
H	5.97436000	-0.32217700	0.22535200
N	6.01407000	1.55322000	-0.67022800
C	4.90016900	1.33390500	-1.57873700
C	3.52049500	1.52921600	-0.92254700
H	3.39057300	2.56586500	-0.59926100
H	2.71257500	1.26637000	-1.59792300
C	6.30917500	2.95671000	-0.40139000
H	5.51267500	3.50474700	0.12726100
H	6.49654400	3.46539900	-1.34810300
H	7.21651000	3.01715600	0.20131400
C	-2.85830800	-0.87035200	-0.03766900
C	-2.51443700	-0.22913300	1.17970400
H	-1.66505100	-0.57779700	1.75403100
C	-3.26686800	0.82026100	1.64123400
H	-3.00340400	1.30854700	2.57290600
C	-4.40613700	1.27418400	0.93047300
C	-5.20464700	2.34779300	1.38988200
H	-4.93463600	2.84595200	2.31412400
C	-6.30745200	2.75146500	0.67745200
H	-6.91365000	3.57383300	1.03733300
C	-6.66242000	2.10039900	-0.52432200
C	-5.90880400	1.05466900	-0.99655100
H	-6.18049700	0.55144800	-1.91753100
C	-4.76525400	0.61556900	-0.28570000
C	-3.96482000	-0.45618500	-0.74771500
H	-4.23423200	-0.95832100	-1.66957300
H	-0.22361200	-2.63592700	0.88788300
H	0.85332700	-3.37971900	2.94465500
H	2.25172800	-2.53069300	3.51687900
H	-7.53644100	2.43035100	-1.07230900

WA-22_H$^{+\prime}$ (aqueous solution)

H	-4.72431700	-0.49691900	-1.99373300

H	-4.57168000	-2.19642300	-2.46207200
C	0.44257000	1.87626900	-1.41046300
S	2.03207000	2.44384900	-0.62258200
C	0.67405900	0.73781000	-2.39712800
H	1.39815800	1.05848500	-3.14602600
H	-0.26251500	0.48397700	-2.89467400
H	1.05063400	-0.15466500	-1.89851200
C	-0.07915000	3.13350900	-2.13956700
H	-0.19994600	3.97844200	-1.46003600
H	-1.04530400	2.91162600	-2.59618400
H	0.62640000	3.41003900	-2.92305700
C	-0.57577900	1.49822500	-0.35003300
N	-0.63510400	2.27579300	0.76878700
C	-1.60040100	2.02885900	1.71019400
N	-2.46381500	1.04714100	1.55277400
C	-2.34455600	0.30360100	0.43385100
N	-1.39971500	0.51890400	-0.54413300
N	-1.64496500	2.79893200	2.79285600
N	-3.18707600	-0.70903200	0.24803900
C	-4.25086800	-1.04172600	1.20213800
H	-4.22143600	-0.33236800	2.02306900
H	-4.04638300	-2.04262200	1.59210600
C	-5.60706000	-1.00335200	0.47997000
H	-6.38852600	-1.31643900	1.17479300
H	-5.81172000	0.02935200	0.18128600
N	-5.66307900	-1.84363400	-0.71413000
C	-4.56063800	-1.50996000	-1.61347600
C	-3.17705800	-1.56195900	-0.94646100
H	-2.93795400	-2.57977500	-0.62632200
H	-2.40156500	-1.21473400	-1.62128500
C	-5.77017800	-3.27193900	-0.42240100
H	-4.90889000	-3.69892200	0.11293100
H	-5.88437700	-3.81554800	-1.36201600
H	-6.66084800	-3.44139700	0.18534000
C	2.79337800	0.95600700	-0.00678000
C	2.47386400	0.45377300	1.28090500
H	1.73614800	0.96284100	1.88720900
C	3.10757500	-0.66335200	1.76329500
H	2.86065500	-1.04443300	2.74788600
C	4.09764700	-1.32998600	0.99841500
C	4.77092500	-2.48250400	1.47256100
H	4.51669200	-2.87152500	2.45214300
C	5.73009800	-3.09777200	0.70463800
H	6.23880100	-3.97933900	1.07608700
C	6.06143100	-2.58870700	-0.57164400
C	5.42790400	-1.47187700	-1.05938800
H	5.67927400	-1.07796500	-2.03774300
C	4.43298400	-0.81523300	-0.29191300
C	3.76012100	0.33561500	-0.76903500
H	4.01508600	0.72840300	-1.74621100
H	0.09926300	2.96604400	0.90449300
H	-1.01961000	3.57417600	2.93695000
H	-2.35414100	2.61487000	3.48405100
H	6.82005600	-3.08532600	-1.16459200

WA-22_2H$^+$ (gas phase)

H	-2.87113500	-2.16438600	-2.41973600
H	-1.70051400	-3.48506000	-2.19996900
C	0.23655600	2.78098800	-1.14759900
S	1.51963000	2.86077500	0.23855100
C	0.71789500	1.99761800	-2.35976600
H	1.58916900	2.49749700	-2.78161200
H	-0.06592400	1.96668000	-3.12132100
H	0.98927600	0.97785300	-2.09569500
C	-0.01901100	4.25435400	-1.49070900
H	-0.16912200	4.88510200	-0.60863100
H	-0.88169900	4.35308400	-2.15372300
H	0.85669600	4.64998700	-2.00292300
C	-0.91253900	2.12228900	-0.45943400
N	-1.71445600	2.88459700	0.35680000
C	-2.72370400	2.30773800	1.07066700
N	-2.96705200	1.00907100	0.97029200
C	-2.16489900	0.32629700	0.14280300
N	-1.15093100	0.84055200	-0.59295300
N	-3.47052000	3.06358500	1.86933500
N	-2.39027200	-1.00386500	0.01364500
C	-3.39865000	-1.70389600	0.79945800
H	-4.04669900	-0.97327100	1.27766600
H	-2.91361700	-2.28658000	1.58958000
C	-4.24621700	-2.59157600	-0.10092800
H	-4.94083100	-3.19669900	0.48029700
H	-4.80371600	-1.99270700	-0.82128800
N	-3.38644700	-3.54289500	-0.91754200
C	-2.33086300	-2.76139200	-1.68479500
C	-1.51102800	-1.87236900	-0.76154300
H	-0.88812900	-2.46527500	-0.08400000
H	-0.84851000	-1.25561700	-1.36395200
C	-2.82123400	-4.68252900	-0.11160900
H	-2.15281800	-4.29153200	0.64941500
H	-2.27825300	-5.34098600	-0.78630800
H	-3.64828100	-5.21612700	0.35201100
C	2.13173900	1.21801600	0.47483800
C	1.65811900	0.44267300	1.56646900
H	0.88530700	0.84804200	2.20853400
C	2.21914000	-0.77956400	1.83549400
H	1.88249900	-1.35541300	2.69101800
C	3.27249900	-1.29537600	1.03752500
C	3.88369600	-2.54235800	1.30649300
H	3.53446400	-3.12988200	2.14822000
C	4.92107300	-2.99387400	0.52648600
H	5.39037500	-3.94476000	0.74728600
C	5.39580500	-2.22111600	-0.55656900
C	4.82721700	-1.00525600	-0.84282500
H	5.19849400	-0.40383500	-1.66469600
C	3.75531400	-0.51141900	-0.05789600
C	3.16975000	0.75063700	-0.30844000
H	3.56544800	1.36488900	-1.10862700
H	-1.48977300	3.86558400	0.46895500
H	-3.32060600	4.05240600	1.99206100
H	-4.00242000	-3.96319900	-1.61674600

H	-4.21029200	2.62460000	2.39571900
H	6.22033300	-2.58942800	-1.15411900

WA-22_2H$^+$ (aqueous solution)

H	4.75246400	0.56757800	-2.03276700
H	4.50337900	2.28049500	-2.43394500
C	-0.48949600	-1.87802800	-1.41572500
S	-2.07642500	-2.43972400	-0.62038500
C	-0.71414300	-0.72470800	-2.38687700
H	-1.44895400	-1.02774200	-3.13247200
H	0.22081900	-0.47824200	-2.89108400
H	-1.07480200	0.16681300	-1.87504900
C	0.01672600	-3.13199000	-2.16287300
H	0.12941100	-3.98679000	-1.49443400
H	0.98360400	-2.91575400	-2.62049500
H	-0.69587000	-3.38871600	-2.94652500
C	0.54202300	-1.52623300	-0.36041000
N	0.59095500	-2.30211900	0.75664900
C	1.57206500	-2.09181400	1.68820700
N	2.45942800	-1.12553900	1.52292500
C	2.33858900	-0.38284700	0.41499500
N	1.39115600	-0.56456400	-0.55739400
N	1.61739300	-2.86519200	2.76324800
N	3.20904400	0.61820600	0.22512400
C	4.25221800	0.94337700	1.19396900
H	4.26866100	0.18200200	1.96779900
H	4.01528600	1.90142700	1.66320300
C	5.61048900	0.97311000	0.51195700
H	6.38470800	1.30759000	1.19852700
H	5.86975200	-0.00979300	0.12274900
N	5.61500300	1.91452800	-0.67379100
C	4.50066600	1.54720800	-1.63081100
C	3.15406200	1.51481700	-0.92685100
H	2.85572500	2.50987500	-0.58821800
H	2.40444700	1.15429100	-1.62396600
C	5.60692600	3.36406700	-0.28159300
H	4.69645500	3.58699200	0.26465600
H	5.65658100	3.95894700	-1.18932100
H	6.47596400	3.54786500	0.34388300
C	-2.82860300	-0.94932100	0.00056600
C	-2.51052900	-0.45685900	1.29225400
H	-1.78100400	-0.97582600	1.90010200
C	-3.13617700	0.66386100	1.77683400
H	-2.89079100	1.03773300	2.76454400
C	-4.11568100	1.34375300	1.01001100
C	-4.77947100	2.50100400	1.48592700
H	-4.52614800	2.88323300	2.46838900
C	-5.72852200	3.12927100	0.71596600
H	-6.22996800	4.01442300	1.08872300
C	-6.05899700	2.62904600	-0.56402800
C	-5.43465100	1.50788600	-1.05358100
H	-5.68533800	1.12063800	-2.03474800
C	-4.45004800	0.83809600	-0.28418300
C	-3.78638600	-0.31712500	-0.76332100
H	-4.04101400	-0.70337700	-1.74315600

H	-0.16081200	-2.97556600	0.89215900
H	0.97212900	-3.62198400	2.92047100
H	6.49211500	1.75281300	-1.17101600
H	2.33849200	-2.69592200	3.44658100
H	-6.80969900	3.13590700	-1.15836200

PPK-32 (gas phase)

H	3.30818100	1.60326000	-2.41000200
H	2.48563100	3.15901700	-2.22543900
C	-0.65101000	-2.58090700	-1.14990300
Se	-2.18977500	-2.38906300	0.16303000
C	-0.90659600	-1.71704100	-2.37646300
H	-1.82643000	-2.03777200	-2.86825100
H	-0.07520600	-1.82717900	-3.08058900
H	-0.98421700	-0.66309800	-2.11724900
C	-0.67864400	-4.06972300	-1.49464500
H	-0.54136300	-4.68743300	-0.60896500
H	0.13603800	-4.29401800	-2.18933800
H	-1.62436000	-4.32365500	-1.97714500
C	0.60488400	-2.18881700	-0.40222900
N	1.18995000	-3.12675200	0.34722400
C	2.27901600	-2.69660300	1.00511700
N	2.79046400	-1.46318800	0.97113600
C	2.13054300	-0.61602500	0.16376500
N	1.02677700	-0.93576500	-0.54880100
N	2.93533600	-3.61717200	1.75764900
N	2.60386000	0.64969200	0.04875000
C	3.76673100	1.12040500	0.79309100
H	4.22907800	0.26572000	1.28035100
H	3.43447200	1.82086700	1.57035100
C	4.74374300	1.80933900	-0.16848600
H	5.57774600	2.23695700	0.39415400
H	5.14890500	1.05436300	-0.85039600
N	4.13533400	2.86275700	-0.97968600
C	2.96089400	2.34061200	-1.67865200
C	1.93634900	1.66828400	-0.75683400
H	1.47621400	2.40681700	-0.08840400
H	1.14546400	1.18741000	-1.32603600
C	3.88356300	4.09735500	-0.24814800
H	3.15831300	4.01378500	0.57729400
H	3.50996600	4.85078600	-0.94524400
H	4.82593700	4.45848800	0.17009700
C	-2.25060100	-0.49192800	0.47107100
C	-1.53366400	0.06760800	1.55814800
H	-0.91825000	-0.57938500	2.17018900
C	-1.62535800	1.40821200	1.83393800
H	-1.07791600	1.83059100	2.67013800
C	-2.43296800	2.26325200	1.04376700
C	-2.54914000	3.65092600	1.30137200
H	-1.99818400	4.07576700	2.13370600
C	-3.34551100	4.44773400	0.51561500
H	-3.42618300	5.50831500	0.72357500
C	-4.06568600	3.89240100	-0.56511000
C	-3.97582200	2.55013800	-0.83964100
H	-4.52883100	2.11948900	-1.66740300

C	-3.16134000	1.70158200	-0.04923400
C	-3.05244200	0.31262900	-0.30717500
H	-3.61707800	-0.11593600	-1.12705700
H	2.45929400	-4.48133300	1.95213000
H	3.63306900	-3.28908100	2.40290600
H	-4.69163400	4.53178500	-1.17647200

PPK-32 (aqueous solution)

H	-3.48764800	1.53199900	2.39628400
H	-2.69818900	3.11324900	2.29192000
C	0.57771200	-2.52712400	1.17823800
Se	2.16750600	-2.41038200	-0.08403100
C	0.81886000	-1.63704700	2.38855400
H	1.71341500	-1.96883300	2.91780600
H	-0.03579800	-1.70891700	3.06850400
H	0.93461000	-0.59239700	2.10722000
C	0.55715000	-4.00497200	1.57076400
H	0.44692100	-4.65187200	0.70218600
H	-0.29028500	-4.18635000	2.23805900
H	1.47411400	-4.26346200	2.10318700
C	-0.64753900	-2.12938300	0.37900900
N	-1.17976600	-3.05528400	-0.42488800
C	-2.25418500	-2.62850300	-1.11659600
N	-2.79281000	-1.40599300	-1.05809200
C	-2.18257600	-0.56664500	-0.20286100
N	-1.09657500	-0.88986900	0.54302200
N	-2.85736500	-3.53297600	-1.92292200
N	-2.68180200	0.68285500	-0.07033000
C	-3.83277100	1.15802900	-0.83554100
H	-4.26362500	0.31697900	-1.37127900
H	-3.48882300	1.89529600	-1.57034100
C	-4.85895800	1.78418600	0.11779200
H	-5.68249900	2.20877100	-0.46080500
H	-5.26516200	0.99376800	0.75740200
N	-4.30635000	2.82495800	0.98734600
C	-3.13915900	2.30523100	1.70373200
C	-2.06978300	1.69411400	0.79016200
H	-1.61677300	2.46690800	0.15822600
H	-1.28343400	1.22216900	1.37189100
C	-4.04948700	4.08574500	0.29461600
H	-3.29533700	4.02947000	-0.50452700
H	-3.71091600	4.82556200	1.02298000
H	-4.98201700	4.44034300	-0.14937900
C	2.30696000	-0.52389200	-0.43356300
C	1.61812800	0.04360000	-1.53529500
H	0.97915400	-0.58505800	-2.14238200
C	1.76309900	1.37597500	-1.83147400
H	1.23472900	1.80614200	-2.67536700
C	2.60160100	2.21067600	-1.05018900
C	2.77302900	3.58884400	-1.33130500
H	2.24216500	4.02138600	-2.17219400
C	3.59725600	4.36588700	-0.55319600
H	3.72049900	5.41891800	-0.77698600
C	4.28945800	3.80056200	0.54188900
C	4.14533800	2.46730800	0.83992200

H	4.67525500	2.02990700	1.67889800
C	3.30151500	1.63925600	0.05704500
C	3.13464500	0.26023600	0.33952600
H	3.67301600	-0.17225200	1.17471900
H	-2.37368400	-4.39215300	-2.12366900
H	-3.54999000	-3.21056100	-2.57785300
H	4.93602100	4.42564500	1.14624200

PPK-32_H$^+$ (gas phase)

H	2.55279700	-2.26629300	-2.50724900
H	3.57035700	-0.85303300	-2.11735000
C	-2.93798700	0.18801100	-1.09303700
Se	-2.84260800	1.69262000	0.27390000
C	-2.31599800	0.62536800	-2.41277300
H	-2.85665800	1.48645600	-2.80622700
H	-2.38621800	-0.19064000	-3.14013300
H	-1.26507900	0.88341800	-2.29783600
C	-4.44081900	-0.04945800	-1.22899500
H	-4.89197200	-0.33087900	-0.27972200
H	-4.61612000	-0.85988800	-1.94174800
H	-4.92582300	0.85091300	-1.60966700
C	-2.21566100	-0.97092000	-0.45798400
N	-2.91121400	-1.81587500	0.29651900
C	-2.19182300	-2.79607800	0.86774700
N	-0.85819300	-2.95851800	0.75006200
C	-0.28914400	-2.05791800	-0.04414300
N	-0.89544000	-1.06154700	-0.68971100
N	-2.85925300	-3.68496300	1.62140500
N	1.08064300	-2.17197600	-0.24484500
C	1.87378800	-3.02657000	0.61127400
H	1.24076800	-3.83339100	0.97794400
H	2.25024500	-2.48300500	1.48953300
C	3.01791200	-3.62536800	-0.18839100
H	3.70139900	-4.19960700	0.43665000
H	2.63375300	-4.25533200	-0.99058600
N	3.84282400	-2.54120600	-0.86996600
C	2.93957300	-1.63937400	-1.70380200
C	1.80754200	-1.08067300	-0.86410200
H	2.17983500	-0.36665700	-0.11834700
H	1.12108800	-0.53610400	-1.50997900
C	4.71277100	-1.77365000	0.08225800
H	4.08604500	-1.23386900	0.78482000
H	5.31366600	-1.07211200	-0.49251100
H	5.35159300	-2.47895100	0.60975100
C	-0.95412700	1.91358700	0.56891200
C	-0.39295900	1.41645600	1.77254600
H	-1.03283200	0.89392500	2.47270400
C	0.93580600	1.61236600	2.05573100
H	1.35148900	1.25173500	2.99115300
C	1.78144700	2.29761500	1.14768900
C	3.15933200	2.51045200	1.40336300
H	3.57955300	2.15126300	2.33730000
C	3.94589600	3.18646400	0.50146600
H	4.99392700	3.35948000	0.71671200
C	3.38905300	3.68610000	-0.69749700

C	2.05568100	3.50265600	-0.97099100
H	1.62256400	3.90016700	-1.88232600
C	1.21762100	2.80519400	-0.06426000
C	-0.16366800	2.61214300	-0.31668700
H	-0.59539400	3.03493200	-1.21612000
H	-3.84935600	-3.57056700	1.75310700
H	4.46854100	-3.01605200	-1.52171900
H	-2.36687200	-4.42589000	2.08777300
H	4.01637600	4.23107500	-1.39270100

PPK-32_H$^+$ (aqueous solution)

H	3.15349900	-1.81603400	2.39253400
H	2.26213400	-3.32848700	2.10301300
C	-0.52929400	2.58292400	1.14831400
Se	-2.05543800	2.49165800	-0.19398400
C	-0.83982500	1.70075900	2.34855200
H	-1.75136900	2.04880000	2.83640200
H	-0.01551700	1.76233000	3.06623300
H	-0.96018200	0.65730300	2.06511800
C	-0.49865800	4.06123800	1.53607700
H	-0.35402500	4.70533200	0.67033600
H	0.33082700	4.23391500	2.22749600
H	-1.42821500	4.33252400	2.03891000
C	0.71745700	2.15528600	0.40376500
N	1.35283200	3.08123000	-0.31575200
C	2.44611000	2.63174600	-0.96259800
N	2.91123700	1.37372900	-0.93037700
C	2.19417000	0.54373700	-0.16600200
N	1.08714400	0.87998500	0.52636400
N	3.13840600	3.52790200	-1.69031500
N	2.61850600	-0.74831400	-0.06712700
C	3.77786900	-1.24458700	-0.78815700
H	4.30712500	-0.40162600	-1.22413500
H	3.46263200	-1.90317100	-1.60424900
C	4.71699500	-1.96466700	0.16597100
H	5.54583500	-2.43159400	-0.36199900
H	5.10344700	-1.27816400	0.91719700
N	3.99561000	-3.05903900	0.92761700
C	2.77568800	-2.49440800	1.62935800
C	1.86854300	-1.76145400	0.65522500
H	1.40371200	-2.45868500	-0.04957900
H	1.07642600	-1.27457400	1.21729500
C	3.68484200	-4.25936700	0.08350900
H	2.99714200	-3.97709200	-0.70673800
H	3.23479900	-5.01324500	0.72377700
H	4.61604000	-4.62536400	-0.34013600
C	-2.26794900	0.60312100	-0.48988600
C	-1.59813800	-0.02069300	-1.57261400
H	-0.93438200	0.56619600	-2.19470000
C	-1.79230000	-1.35471200	-1.83096800
H	-1.27954000	-1.82831500	-2.66108700
C	-2.66049100	-2.13544900	-1.02674000
C	-2.87973900	-3.51466600	-1.26644600
H	-2.36363000	-3.99058500	-2.09295800
C	-3.73110100	-4.23855000	-0.46661800

H	-3.89088600	-5.29292900	-0.65864900
C	-4.40428800	-3.61658700	0.60953700
C	-4.21421900	-2.28075600	0.86729400
H	-4.72916400	-1.79970400	1.69154000
C	-3.34102200	-1.50687400	0.06147700
C	-3.12669500	-0.12672800	0.30243900
H	-3.65292900	0.34966300	1.12126500
H	2.76871300	4.45459200	-1.81506900
H	4.63261300	-3.37363900	1.66052600
H	3.90864100	3.22214000	-2.25988600
H	-5.07273400	-4.20028700	1.23119800

PPK-32_H$^{+\prime}$ (gas phase)

H	4.91294700	0.32974100	-1.97473200
H	4.81764100	1.98400200	-2.58841700
C	-0.32524800	-1.87388700	-1.29626300
Se	-2.06962300	-2.34389100	-0.39038400
C	-0.53079300	-0.82505200	-2.38057000
H	-1.25104600	-1.19808100	-3.10846300
H	0.41337300	-0.62616500	-2.89115800
H	-0.90047100	0.11264500	-1.96681100
C	0.14735600	-3.21315800	-1.89830500
H	0.25992300	-3.99217500	-1.14161200
H	1.10902000	-3.07263900	-2.39759400
H	-0.57949400	-3.55246500	-2.63660400
C	0.68542000	-1.42720700	-0.26637000
N	0.70490700	-2.10395600	0.92632600
C	1.64600600	-1.78068000	1.86529600
N	2.53848400	-0.84580200	1.64618800
C	2.46535400	-0.20144400	0.45639800
N	1.54293300	-0.49200200	-0.52378100
N	1.62692400	-2.43606000	3.03248200
N	3.33096700	0.77421800	0.20876600
C	4.38477800	1.16462000	1.15561800
H	4.31464300	0.53547400	2.03753500
H	4.19948100	2.20310400	1.44428200
C	5.75553600	1.02885100	0.46516600
H	6.53243500	1.38072200	1.14624700
H	5.94004800	-0.03172600	0.26517200
N	5.85004700	1.75716700	-0.78936200
C	4.76887500	1.37549900	-1.68362500
C	3.36926500	1.52057800	-1.05732100
H	3.15686200	2.56826600	-0.82652700
H	2.59288600	1.13937400	-1.71298100
C	6.03407800	3.19706200	-0.64157100
H	5.19069600	3.72600000	-0.16913300
H	6.19617800	3.63500600	-1.62751400
H	6.92533600	3.37881700	-0.03925200
C	-2.71546100	-0.61662100	0.15645600
C	-2.36666100	-0.07640400	1.41873300
H	-1.71344900	-0.63001500	2.08185600
C	-2.87837900	1.13479300	1.81108200
H	-2.61747500	1.54591500	2.78006700
C	-3.76444000	1.86068600	0.97679400
C	-4.31159100	3.10806500	1.36054100

H	-4.04003100	3.52722600	2.32277900
C	-5.17699800	3.77587500	0.52925100
H	-5.59229700	4.72924300	0.83246300
C	-5.53626600	3.22773500	-0.72187700
C	-5.02414700	2.01924200	-1.12330200
H	-5.30132000	1.59425100	-2.08138100
C	-4.12790300	1.30646100	-0.28945800
C	-3.58524400	0.05622200	-0.67316900
H	-3.87053300	-0.36977500	-1.62792400
H	-0.10563100	-2.69669900	1.11652300
H	1.02237700	-3.22187200	3.20233700
H	2.33659000	-2.21477600	3.71215400
H	-6.22258000	3.76652500	-1.36358800

PPK-32_H$^{+\prime}$ (aqueous solution)

H	4.41557400	0.52725500	-2.14436600
H	4.10591500	2.20932200	-2.59814900
C	-0.55310800	-2.20224900	-1.29792700
Se	-2.32739000	-2.30856000	-0.33002200
C	-0.64209700	-1.24391400	-2.47681600
H	-1.41793100	-1.59027800	-3.15940000
H	0.31187400	-1.22028800	-3.00836000
H	-0.87621900	-0.23007200	-2.15554200
C	-0.29989600	-3.64272300	-1.77438000
H	-0.28407300	-4.36542600	-0.95739800
H	0.65996200	-3.68755400	-2.29390000
H	-1.08483600	-3.93283300	-2.47320800
C	0.49895100	-1.77478100	-0.30676000
N	0.65920300	-2.53116500	0.82182400
C	1.63670900	-2.20129500	1.72304700
N	2.42696000	-1.16736900	1.52020200
C	2.21154000	-0.44950000	0.39933800
N	1.24859900	-0.74401700	-0.53888800
N	1.76966300	-2.94580400	2.81674800
N	2.97225700	0.61760900	0.16952100
C	4.05127400	1.03157300	1.07332000
H	4.11713700	0.32035800	1.89054000
H	3.78853900	2.01241700	1.47912500
C	5.36994400	1.10040400	0.28632200
H	6.15718400	1.47230400	0.94466800
H	5.63957100	0.08727700	-0.02733900
N	5.30294600	1.94389700	-0.90470800
C	4.18963500	1.52387400	-1.75279400
C	2.83958100	1.46524600	-1.02140500
H	2.53502500	2.46103000	-0.68753900
H	2.06353200	1.05642500	-1.66006300
C	5.31023900	3.37582700	-0.61142200
H	4.44332400	3.73286700	-0.03560000
H	5.33837400	3.92786000	-1.55261700
H	6.21150000	3.61429500	-0.04373500
C	-2.57712600	-0.48064200	0.21892600
C	-2.11287000	-0.05159700	1.48739600
H	-1.59764700	-0.75052300	2.13393800
C	-2.32695400	1.24100300	1.89708900
H	-1.97166800	1.56844800	2.86784100

C	-3.01574900	2.16462600	1.07133800
C	-3.25106900	3.50422800	1.46807800
H	-2.88845000	3.83532700	2.43477300
C	-3.92674400	4.37020900	0.64253000
H	-4.10064400	5.39295900	0.95509100
C	-4.39995800	3.93714800	-0.61708900
C	-4.18941000	2.64441900	-1.03049800
H	-4.55094400	2.30828400	-1.99585600
C	-3.49432900	1.72722900	-0.20237900
C	-3.26111000	0.38813400	-0.60245200
H	-3.63101400	0.05604600	-1.56522300
H	-0.01277200	-3.26366500	1.01949500
H	1.20116400	-3.75652900	2.99669700
H	2.48769000	-2.70183100	3.47956500
H	-4.93103600	4.63220400	-1.25632200

PPK-32_2H$^+$ (gas phase)

H	2.82480500	2.36213300	-2.40663700
H	1.65411500	3.66066700	-2.07983700
C	-0.16450800	-2.65980900	-1.19908200
Se	-1.52265100	-2.67363000	0.33862300
C	-0.66722000	-1.87022500	-2.39685300
H	-1.56442700	-2.34759900	-2.78935200
H	0.09246200	-1.86334600	-3.18434200
H	-0.90089100	-0.84158100	-2.13128700
C	0.02848200	-4.14376900	-1.52088800
H	0.18573100	-4.76809700	-0.63499700
H	0.86746800	-4.28335300	-2.20821600
H	-0.87178900	-4.52114400	-2.00361200
C	0.99242100	-2.02224500	-0.52554100
N	1.84695100	-2.80739800	0.21559600
C	2.87819300	-2.24454200	0.90949100
N	3.10075300	-0.94037200	0.85398500
C	2.25627300	-0.23625900	0.08623700
N	1.21736000	-0.73136400	-0.62145300
N	3.66935200	-3.02279900	1.64205700
N	2.46771300	1.10051100	-0.00295600
C	3.50464200	1.77859300	0.76373000
H	4.18035500	1.03615500	1.18165800
H	3.05250000	2.32438100	1.59857800
C	4.30202700	2.71028700	-0.13793300
H	5.01782600	3.29915600	0.43423700
H	4.83014500	2.14642400	-0.90703100
N	3.39701000	3.68449800	-0.87498100
C	2.31370800	2.92330600	-1.62413900
C	1.54572700	1.98942700	-0.70070600
H	0.94851000	2.54773800	0.02746000
H	0.86154100	1.38980600	-1.29611800
C	2.85957000	4.78410300	0.00199200
H	2.23085600	4.35502800	0.77617400
H	2.27973600	5.46327900	-0.61939400
H	3.70227500	5.30756900	0.44865200
C	-2.04520700	-0.84420600	0.52927600
C	-1.52686800	-0.08057800	1.60682900
H	-0.79657200	-0.52355700	2.27293100

C	-1.99432800	1.19024500	1.82990800
H	-1.62843100	1.76188100	2.67641800
C	-2.98798100	1.76476300	0.99626800
C	-3.50259300	3.06386300	1.21827300
H	-3.12548600	3.64458100	2.05276500
C	-4.48486700	3.57432100	0.40402400
H	-4.88172300	4.56486500	0.58989400
C	-4.99834400	2.81148200	-0.66841500
C	-4.52372200	1.54644900	-0.90933400
H	-4.92642600	0.95313300	-1.72227100
C	-3.51102900	0.99100000	-0.08784300
C	-3.02595100	-0.32253900	-0.29002000
H	-3.45653500	-0.92401700	-1.08183100
H	1.65159800	-3.79806500	0.28618900
H	3.53422300	-4.01694900	1.73392700
H	3.97620600	4.13925300	-1.58391800
H	4.42667200	-2.59540500	2.15263900
H	-5.77910900	3.22693500	-1.29322900

PPK-32_2H^{+} (aqueous solution)

H	-3.36725700	1.51537200	2.38529800
H	-2.58579900	3.10714500	2.26092200
C	0.65055700	-2.51258200	1.21398200
Se	2.16649200	-2.39192600	-0.13961800
C	0.91254300	-1.60409000	2.40623700
H	1.83222900	-1.91761900	2.89955100
H	0.08602500	-1.68595600	3.11775200
H	1.00301800	-0.56210700	2.10741600
C	0.64007700	-3.98931400	1.62228100
H	0.62947900	-4.67792200	0.77370900
H	-0.22974100	-4.19477000	2.25033400
H	1.53984500	-4.20356900	2.19775700
C	-0.56969100	-2.09507400	0.44987100
N	-1.18113100	-3.01199400	-0.35825400
C	-2.28075800	-2.65306000	-1.08951600
N	-2.76673300	-1.42614800	-1.02125200
C	-2.12932400	-0.57752800	-0.20221800
N	-1.03233900	-0.88195100	0.55154500
N	-2.85651300	-3.55839800	-1.86907700
N	-2.60051200	0.67437900	-0.10302500
C	-3.76055200	1.13663000	-0.85909100
H	-4.19474600	0.29680400	-1.39261500
H	-3.43565800	1.87678700	-1.59447400
C	-4.80592800	1.70868900	0.08457600
H	-5.63501100	2.14413800	-0.46811300
H	-5.18096000	0.94198100	0.75984600
N	-4.22216000	2.79726500	0.95976900
C	-3.00547800	2.27723400	1.69726400
C	-1.98079600	1.69633900	0.73596900
H	-1.55013200	2.46945700	0.09521400
H	-1.17857700	1.24595400	1.31181300
C	-3.96240500	4.07492900	0.21536400
H	-3.21054500	3.90330500	-0.54782400
H	-3.61146100	4.81516200	0.92871300
H	-4.89581600	4.39432900	-0.23969300

C	2.29256000	-0.50390500	-0.46820400
C	1.60407700	0.06632700	-1.56836500
H	0.97537400	-0.56080200	-2.18771100
C	1.74252100	1.40281300	-1.84790400
H	1.21690600	1.83860400	-2.69033100
C	2.57105300	2.23365200	-1.05207200
C	2.73332100	3.61590500	-1.31713700
H	2.20292200	4.05421800	-2.15509500
C	3.54936500	4.38917000	-0.52700700
H	3.66621300	5.44535400	-0.73858900
C	4.24231700	3.81697900	0.56410100
C	4.10733600	2.47988600	0.84717600
H	4.63766000	2.03665700	1.68259400
C	3.27164100	1.65563200	0.05170700
C	3.11519800	0.27311700	0.31797900
H	3.65627000	-0.16670900	1.14725800
H	-0.80050100	-3.94595500	-0.43094700
H	-2.52695000	-4.50717800	-1.94172100
H	-4.92541000	3.00910500	1.66933300
H	-3.67061900	-3.28902900	-2.39826900
H	4.88243500	4.44017300	1.17696100

H_2O (aqueous solution)

O	0.00000000	0.00000000	0.11877700
H	0.00000000	0.75947400	-0.47510800
H	0.00000000	-0.75947400	-0.47510800

H_3O^+ (aqueous solution)

O	0.00000000	0.00000000	0.08668300
H	0.00000000	0.92560600	-0.23115400
H	-0.80159800	-0.46280300	-0.23115400
H	0.80159800	-0.46280300	-0.23115400
